# The Relationship between Water, Sanitation and Schistosomiasis: A Systematic Review and Meta-analysis

**DOI:** 10.1371/journal.pntd.0003296

**Published:** 2014-12-04

**Authors:** Jack E. T. Grimes, David Croll, Wendy E. Harrison, Jürg Utzinger, Matthew C. Freeman, Michael R. Templeton

**Affiliations:** 1 Department of Civil and Environmental Engineering, Imperial College London, London, United Kingdom; 2 Department of Epidemiology and Public Health, Swiss Tropical and Public Health Institute, Basel, Switzerland; 3 University of Basel, Basel, Switzerland; 4 Schistosomiasis Control Initiative, Imperial College London, London, United Kingdom; 5 Department of Environmental Health, Rollins School of Public Health, Emory University, Atlanta, Georgia, United States of America; Brown University, United States of America

## Abstract

**Background:**

Access to “safe” water and “adequate” sanitation are emphasized as important measures for schistosomiasis control. Indeed, the schistosomes' lifecycles suggest that their transmission may be reduced through safe water and adequate sanitation. However, the evidence has not previously been compiled in a systematic review.

**Methodology:**

We carried out a systematic review and meta-analysis of studies reporting schistosome infection rates in people who do or do not have access to safe water and adequate sanitation. PubMed, Web of Science, Embase, and the Cochrane Library were searched from inception to 31 December 2013, without restrictions on year of publication or language. Studies' titles and abstracts were screened by two independent assessors. Papers deemed of interest were read in full and appropriate studies included in the meta-analysis. Publication bias was assessed through the visual inspection of funnel plots and through Egger's test. Heterogeneity of datasets within the meta-analysis was quantified using Higgins' *I^2^*.

**Principal Findings:**

Safe water supplies were associated with significantly lower odds of schistosomiasis (odds ratio (OR) = 0.53, 95% confidence interval (CI): 0.47–0.61). Adequate sanitation was associated with lower odds of *Schistosoma mansoni*, (OR = 0.59, 95% CI: 0.47–0.73) and *Schistosoma haematobium* (OR = 0.69, 95% CI: 0.57–0.84). Included studies were mainly cross-sectional and quality was largely poor.

**Conclusions/Significance:**

Our systematic review and meta-analysis suggests that increasing access to safe water and adequate sanitation are important measures to reduce the odds of schistosome infection. However, most of the studies were observational and quality was poor. Hence, there is a pressing need for adequately powered cluster randomized trials comparing schistosome infection risk with access to safe water and adequate sanitation, more studies which rigorously define water and sanitation, and new research on the relationships between water, sanitation, hygiene, human behavior, and schistosome transmission.

## Author Summary

Schistosomiasis is a serious disease in many developing countries, and the control of schistosomiasis relies on the large-scale administration of praziquantel. However, this strategy fails to address the root causes of schistosomiasis, which people acquire during contact with freshwater bodies that contain infected snails. It is suggested that improving access to clean water and sanitation reduces the risk of schistosomiasis transmission. Moreover, the use of soap, detergent, and endod (a berry sometimes used as a substitute for soap) might kill snails and the parasite larvae they excrete. We systematically reviewed the literature and performed a meta-analysis to study the association between people's access to clean water, sanitation, and good hygiene and the risk of schistosomiasis. People with access to clean water and adequate sanitation were at lower risks of schistosomiasis. No studies were found to explore the relationship between hygiene and risk of schistosomiasis. The difference in infection rates between people with and without access to clean water and sanitation varies widely between studies, suggesting that the impact of water and sanitation on schistosomiasis transmission is mediated by many other social and environmental factors. Further research is needed on the impact of water, sanitation and hygiene interventions for schistosomiasis control.

## Introduction

More than 200 million people are estimated to be infected with schistosomes, among about 800 million at risk of schistosomiasis [1]. Three species of schistosome comprise the majority of these infections. Intestinal schistosomiasis is mostly caused by *Schistosoma mansoni* and *Schistosoma japonicum*, and the parasite eggs are released in the feces. In urogenital schistosomiasis, caused by *Schistosoma haematobium*, the eggs are released in the urine [2], [3]. Chronic intestinal schistosomiasis is manifested by debilitating symptoms, such as hepatosplenomegaly (enlargement of the liver and spleen) [2], [3], while *S. haematobium* is associated with an increased risk of developing bladder cancer [4], and thought to exacerbate the transmission of HIV and its progression to AIDS [5]. Both intestinal and urogenital schistosomiasis can cause anemia and malnutrition [6], and occasionally the eggs enter the central nervous system, causing symptoms such as seizures and focal neurological deficits [2], [3], [7].

Access to safe water and adequate sanitation are considered important components of schistosomiasis control, which at present largely relies on preventive chemotherapy with a single drug, praziquantel [8]. Adult schistosomes live within humans and, particularly in the case of *S. japonicum*, other mammals (e.g., water buffaloes) [9]. Aquatic snails (in the case of *S. mansoni* and *S. haematobium*) or amphibious snails (*S. japonicum*) act as intermediate hosts and release cercariae. People become infected during contact with infested water, when these cercariae penetrate through the skin. In turn, snails are infected by miracidia, which are released from eggs in the definitive host's urine or feces [2], [3]. Humans avoiding water contact and preventing urine and feces from entering freshwater bodies should therefore halt schistosome transmission. Furthermore, soap and endod (a natural soap substitute) are toxic to cercariae, miracidia, and snails, suggesting that their use may protect from schistosome infection, and thus implying a possible role for hygiene in schistosomiasis control [10], [11]. However, water, sanitation, and hygiene (WASH) are inadequate in large parts of low- and middle-income countries, where schistosomiasis is endemic [2], [3], [12]. Over the past 20 years, the need for multisectoral and integrated approaches to the control of schistosomiasis and other neglected tropical diseases (NTDs) has been emphasized [13]–[26]. Investigation of such approaches are particularly crucial as countries aim for elimination of schistosomiasis in line with World Health Assembly (WHA) resolution 65.19, put forward in May 2012.

Inadequate WASH is estimated to be responsible for 4.0% of deaths and 5.7% of disease burden worldwide, primarily driven by its role in the transmission of diarrheal disease and helminthiases [27]. The evidence for the impact of integrated control of NTDs is accumulating. In a 1978 study in St. Lucia, *S. mansoni* infection rates fell following the provision of safe water supply [28]. In the People's Republic of China, Wang et al. (2009) found that the integration of improved water and sanitation provision significantly reduced infections with the soil-transmitted helminths (STHs) *Ascaris lumbricoides* and *Trichuris trichiura* in addition to *S. japonicum* [29]. In Ethiopia, King et al. (2013) documented declines in *S. mansoni* and STH prevalences during a trachoma control program, which increased the use of improved water sources and latrines [30]. Asaolu and Ofoezie (2003) found sanitation and health education to be important interventions for the control of schistosomiasis and other helminthiases [31]. In Kenya, Freeman et al. (2013) quantified how a school WASH intervention reduced *A. lumbricoides* infection above provision of mass drug administration alone [32].

Relatively little evidence has been systematically collated and analyzed to inform policy-relevant discussions about the importance of including WASH as a part of schistosomiasis control. A previous review, conducted more than 20 years ago, identified four rigorous studies comparing schistosome infection rates with access to clean water, with a median reduction in schistosome morbidity for people with access to improved water supplies of 77% [33]. Many more relevant studies have been published since, providing the motivation for the current systematic review and meta-analysis.

## Methods

We carried out a systematic review and meta-analysis of studies comparing *Schistosoma* infection rates in people with and without access (defined as the availability or use of) to safe water, adequate sanitation, and good hygiene, according to the ‘Meta-analysis Of Observational Studies in Epidemiology’ (MOOSE) guidelines [34], and the Preferred Reporting Items for Systematic Reviews and Meta-Analyses' (PRISMA) statement [35]. Our protocol is available in Text S1, our MOOSE checklist in Text S2, and our PRISMA checklist in Text S3.

We systematically searched PubMed, Web of Science, Embase, and the Cochrane Library from inception to 31 December 2013. Two sets of search terms were developed: one for the diseases, and one for WASH. The standardized ‘improved’ water and sanitation definitions, developed by the World Health Organization (WHO) and UNICEF Joint Monitoring Programme (JMP) [12], were rarely used in the literature. Furthermore, studies seldom distinguished reliably between availability and use of WASH. Therefore, the categories of WASH ‘availability’ and ‘use’ were combined to form the category of ‘access to’. We considered all types of water in Box 1 as ‘safe’, and all types of sanitation in Box 1 as ‘adequate’. We considered ‘well’ to be a safe water source, except in Brazil, where ‘wells’ often consisted of pond-like water bodies, in contrast to the hand-dug wells in sub-Saharan Africa that are unlikely to contain snails or allow for water contact [36]. We considered use of soap during water contact as ‘good’ hygiene practice.

**Box 1. Our Definitions of Safe Water and Adequate Sanitation**

Safe water sources included those described as ‘closed’ rather than ‘open’, ‘piped water’, ‘drinking water’ or ‘cistern’ in the home, ‘clean’ rather than ‘river or lake’, ‘adequate’, ‘public supplies’, ‘treated’, or ‘safe’. Wells were considered safe except in South America. The category of ‘non-use of water from ponds or irrigation wells’ was also included on the assumption that it refers to the water used for most or all domestic water needs. However, studies reporting use of different water sources for different activities were not included in the meta-analysis since they are not readily comparable.

Adequate forms of sanitation considered included ‘(pit) latrine’, ‘flush toilet’, ‘sewer connection’ or ‘sewerage, ‘cesspool’ or ‘septic tank’. Most studies did not indicate where this sanitation drained to.

Search terms were combined as follows, such that each WASH term would be searched in conjunction with each disease term: (*schistosomiasis* or *schistosome* or *schistosoma* or *bilharzia* or *bilharziasis* or *snail fever*) and (*water* or *borehole* or *standpipe* or *rainwater* or *sanitation* or *sanitary engineering* or *latrine* or *toilet* or *pit* or *open defecation* or *open urination* or *shower* or *laundry* or *hygiene* or *detergent* or *soap* or *risk factor*). We did not use Medical Subject Headings (MeSH) terms since some WASH MeSH terms had been introduced only recently, and hence, relevant literature might have been missed during our search.

We also scanned the bibliographies of previous reviews pertaining to WASH and other NTDs [33], [37]–[39]. Additionally, when any study under consideration cited another which appeared to provide relevant evidence, the second study was eligible for inclusion. If a study demonstrated that eligible data had been collected but not reported, the authors were contacted and kindly asked to provide the data for further analysis.

### Study Selection and Data Extraction

Odds ratios (ORs) with 95% confidence intervals (CIs) for prevalence of *Schistosoma* infection according to availability of WASH were used as summary measures in all meta-analyses. Any paper reporting these directly, or providing data from which an OR with a 95% CI could be calculated (for instance 2×2 tables of numbers of people infected and not infected amongst those with and without access to safe water, adequate sanitation, or good hygiene, or sensitivities, specificities, and positive predictive values of these as diagnostics of *Schistosoma* infection), was eligible for inclusion. The searches were carried out without restrictions on language or year of publication.

Studies returned by the searches were screened independently by two assessors (JETG and DC), and disagreements were discussed until consensus was reached. First, the duplicates were removed. Next, titles, and then abstracts (of the remaining papers; if available) were reviewed in order to exclude papers whose titles or abstracts revealed that they were definitely not about WASH, not about human schistosomiasis, or did not contain data that would qualify for inclusion in the meta-analysis. Papers without abstracts or where abstracts were not available were reviewed in full.

The full texts of the remaining papers were sought from Imperial College London, the Swiss Tropical and Public Health Institute, the London School of Hygiene and Tropical Medicine, and the Wellcome and British Libraries. Those obtained were read by JETG and DC, and papers not reporting prevalence according to availability of water and/or sanitation were excluded. Papers in French, Portuguese, and Chinese were discussed with fluent speakers.

Data (2×2 tables where available, or ORs and corresponding 95% CIs) were extracted independently by JETG and DC from the papers, or (when supplied), from authors' correspondence. Crude or unadjusted ORs from bivariate analyses were taken where available, to minimize the risk of water supplies' impact being reported as due to the water contact they prevent, rather than due to the water supplies *per se*. Discrepancies were discussed and, if needed, a third person (JU) was consulted until consensus was reached. Where studies reported datasets from different settings, all datasets were eligible for inclusion. Where they reported different ORs for different forms of water or sanitation in the same setting, all ORs were included in the meta-analysis (double-counting some participants was felt to be preferable to the bias that would be induced by choosing one of the ORs). Where a 2×2 table contained one or more zeros, a Woolf-Haldane continuity correction was applied and 0.5 was added to all four of that table's elements [40].

### Quality Assessment

Study quality was assessed using a checklist based on the GRADE approach [41] and other recent and similar systematic reviews [37]–[39]. Study assessment considered diagnostics (with sedimentation for intestinal schistosomiasis being rewarded due to its higher sensitivity compared with a single Kato-Katz thick smear reading) [42], method of assessment of WASH, correction for confounders, response rates, and other strengths and weaknesses (see Tables S2, S3, S4).

### Synthesis of Results

*S. haematobium* may be less susceptible than *S. mansoni* and *S. japonicum* to control with sanitation, since urination into water bodies is generally thought to be less easily controlled than open defecation [43], [44]. On the other hand, all human schistosomes infect people during contact with infested water, so we might expect water supplies to have a similar effect on infection with any schistosome species. We therefore pooled different species in the water meta-analysis, but carried out species-specific analyses for sanitation. The effect of species was subsequently investigated in the water sub-analyses. No studies reported data eligible for an analysis of hygiene and schistosomiasis.

The impact of WASH on schistosomiasis is likely to be mediated by a number of other factors, including behavioral and environmental ones, and aspects related to socioeconomic status (SES), which may vary between study settings. It is therefore reasonable to expect some variability in the true effect size between studies. Hence, random effects models [45] in StatsDirect version 2.8.0 (StatsDirect Ltd, Altrincham, United Kingdom) were employed in all the meta-analyses. These models weighted datasets' effect sizes by their inverse variances.

### Publication Bias and Sensitivity Analysis

Publication bias was assessed through the visual inspection of funnel plots and through Egger's test [46]. Higgins' *I^2^* was used to assess heterogeneity between studies [47]. Where heterogeneity was high (*I^2^*>75%) and a meta-analysis included at least one study of a different age group (adults, children, or mixed, with children defined as those below 18 years of age, or attending school), from a different continent, with a different schistosome species, with water in a different location, or with a different kind of sanitation, sub-analyses divided the datasets according to these factors to see if this reduced heterogeneity.

Sensitivity analyses were used to check for the impact of the largest studies on the three meta-analyses. All datasets from the study contributing the greatest weight to each meta-analysis was removed, and the effect on the results was investigated.

## Results

### Study Selection

**Figure 1:**
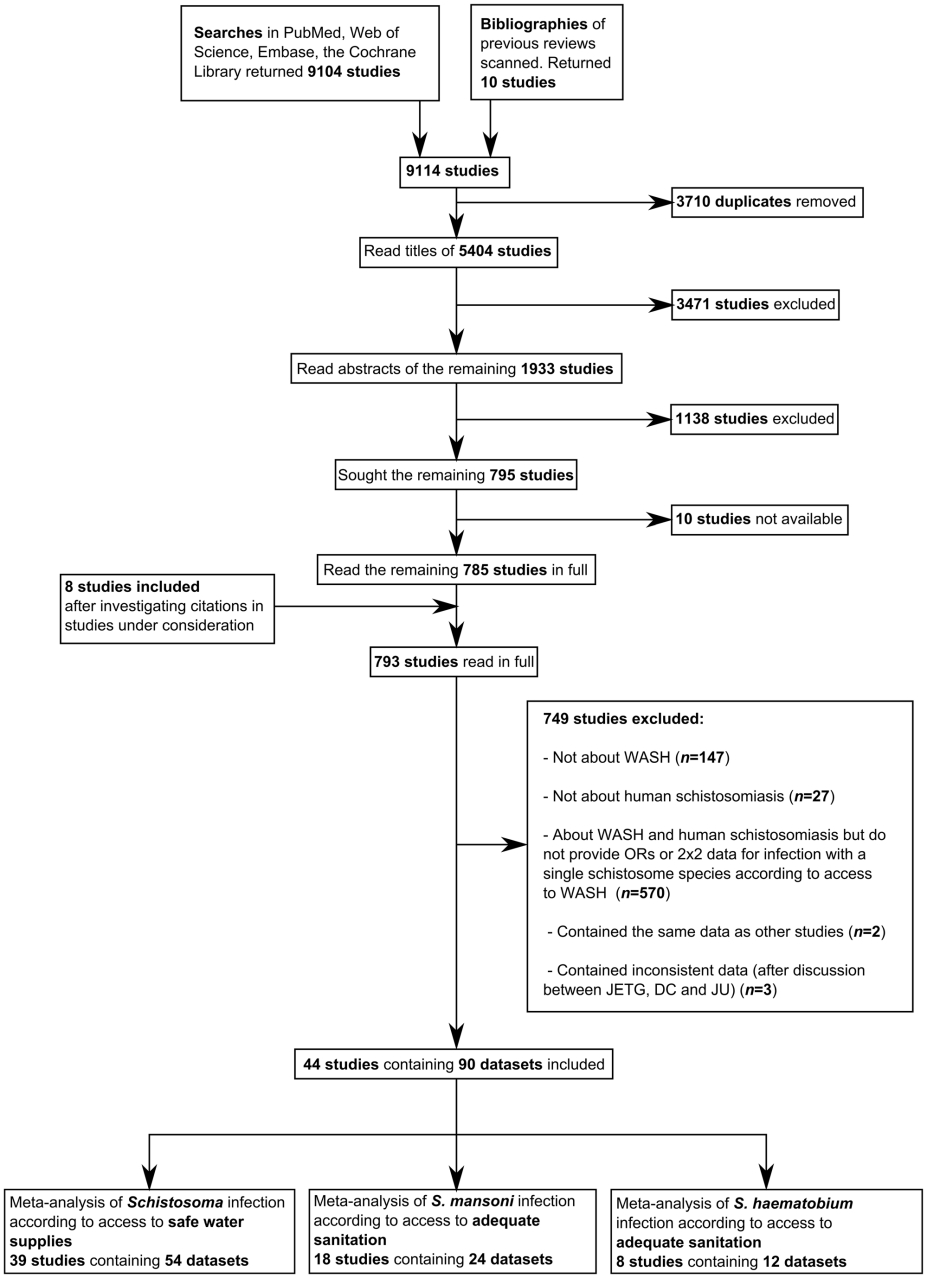
Flow diagram demonstrating identification, inclusion and exclusion of studies.

The searches and bibliographies of previous reviews returned 9,114 studies, 5,404 of which were unique (Figure 1). Finally, 44 relevant studies containing 90 datasets were identified. These 90 datasets consist of 54 datasets comparing safe water with schistosomiasis (35 on *S. mansoni*, 17 on *S. haematobium*, and two on *S. japonicum*), 24 comparing adequate sanitation with *S. mansoni*, and 12 comparing adequate sanitation with *S. haematobium*. No eligible studies compared sanitation with *S. japonicum*, or hygienic practices with *Schistosoma* infection rates, so meta-analyses were not conducted for these associations. A number of studies discussed related topics such as the survival of free-living schistosome stages, or the relationship between water supplies and water contact. However, these did not meet the inclusion criteria and were therefore excluded from the current review. The full list of excluded papers (along with reasons for exclusion) is found in Table S1.

Safe water sources were most commonly described as ‘tap’ or ‘piped’ (24 datasets), followed by ‘borehole’, ‘well’, or ‘standpipe’ (18 datasets), and ‘not using environmental water bodies such as rivers and lakes’ (four datasets), then by ‘adequate source of drinking water’ (three datasets), not using ‘unsafe’ water, and ‘domestic drinking water’ (two datasets each). The remaining dataset referred to ‘clean household water’. In the sanitation and *S. mansoni* analysis, adequate sanitation was mostly described as a ‘latrine’ (12 datasets), followed by ‘latrine or flush toilet’ (six datasets). Two datasets referred to each of ‘septic tank’ or ‘cesspool’, ‘sewer connection’ or ‘latrine’, and ‘sewerage’. In the sanitation and *S. haematobium* analysis, adequate sanitation was most commonly described as a ‘latrine’ (eight datasets), followed by ‘latrine’ or ‘flush toilet’ (two datasets), and finally by ‘septic tank’ or ‘cesspool’, then ‘toilet’ (one dataset each).

Studies most commonly included children and adults (21 studies). Another 19 studies included only children (i.e. individuals below the age of 18 years, or in school), while four studies were of adults only. The included studies were most commonly from Africa (21 studies). Another 17 studies were from Brazil. The remaining six studies were from Asia (four in Yemen and two in the People's Republic of China). The most common language was English (40 studies), and the remaining four studies were in Portuguese. Three studies had case-control designs, while the remaining 41 contained descriptive cross-sectional data. Study quality was generally low, with water and sanitation rarely being defined in a uniform way, or assessed through inspections. Furthermore, very few studies provided data split according to confounders such SES. Of the 21 studies whose authors were contacted, data were only provided for the studies by Knopp et al. (2013) [48], [49], Arndt et al. (2013) [50], Fürst et al. (2013) [51], and Sady et al. (2013) [52].

### Water

#### Results of individual studies and synthesis of results

**Figure 2:**
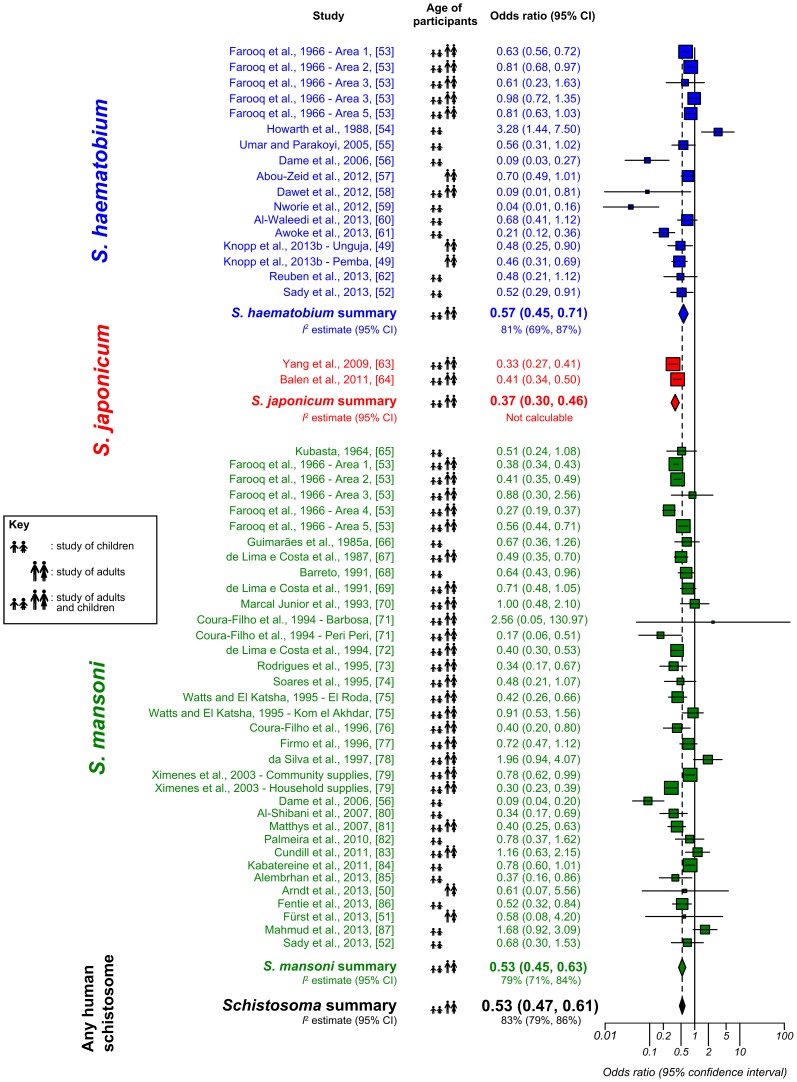
Forest plot for *Schistosoma* infection according to availability or use of a safe water source. Studies on *S. haematobium* are grouped at the top in blue, followed by those on *S. japonicum* in red, and those on *S. mansoni* in green. The square sizes represent the weight given to each dataset, and the black horizontal lines represent 95% confidence intervals. For each species the rhombus is centred on the combined effect size, and its width represents the 95% confidence interval. *I^2^* estimates are presented beneath each combined effect size (except for *S. japonicum*, since two studies is insufficient for the *I^2^* calculation). The combined effect size for all human schistosome species is presented at the bottom.

We found that access to safe water sources was associated with a significantly lower odds of schistosome infection (OR = 0.53, 95% CI: 0.47–0.61). This association held for *S. haematobium* (OR = 0.57, 95% CI: 0.45–0.71), for *S. japonicum* (OR = 0.37, 95% CI: 0.30–0.46), and for *S. mansoni* (OR = 0.53, 95% CI: 0.45–0.63). The details of the studies comparing access to safe water supplies with *Schistosoma* infection, along with their quality assessment scores, are shown in Table S2. The respective forest plot is given in Figure 2, in which the symbol next to each study's effect size denotes whether the participants were children (<18 years of age or in school), adults, or a combination. Of the 54 datasets to report *Schistosoma* infection according to access to safe water source, 29 reported infection rates to be significantly lower in those with safe water. Another 24 reported no significant difference, while one dataset found significantly higher infection rates in those with access to safe water.

#### Risk of bias, sub-group analysis, and sensitivity analysis

Publication bias was deemed unlikely, given the symmetrical funnel plot (see Figure S1) and Egger's test (*P* = 0.84). These studies demonstrated high heterogeneity, with a Higgins' *I^2^* value of 83% (95% CI: 79–86%), which was not much reduced by dividing the datasets according to species – for *S. haematobium* (17 datasets) *I^2^* was 81% (95% CI: 69–87%), for *S. mansoni* (35 datasets) it was 79% (95% CI: 71–84%), whilst it could not be calculated for the *S. japonicum* meta-analysis, since only two datasets pertained to this species. Further sub-analyses divided the datasets according to participants' ages (children, adults, or both), location of water source (household, community, or not specified), and continent (Africa, South America, or Asia).

Studies of children (19 datasets, OR = 0.49, 95% CI: 0.35–0.68, *I^2^* = 82%), adults (five datasets, OR = 0.56, 95% CI: 0.44–0.72, *I^2^* = 0%), and studies including both adults and children (30 datasets, OR = 0.54, 95% CI: 0.46–0.63, *I^2^* = 86%) all showed similar effect sizes to the overall water meta-analysis.

Access to a household rather than environmental source such as a river or lake (four datasets, OR = 0.57, 95% CI: 0.28–1.17, *I^2^* = 89%) or a household rather than an undefined source (13 datasets, OR = 0.59, 95% CI: 0.46–0.76, *I^2^* = 64%) also showed similar results to the overall water meta-analysis, as did access to a community rather than environmental source (14 datasets, OR = 0.60, 95% CI: 0.47–0.76, *I^2^* = 91%). A further 23 datasets did not specify if the safe water supplies were available in the household or in the community. These also had a similar OR to the overall meta-analysis (0.45, 95% CI: 0.36–0.56, *I^2^* = 74%).

Water supplies had similar OR for infection in Africa (31 datasets, OR = 0.52, 95% CI: 0.43–0.62, *I^2^* = 86%) and South America (17 datasets, all from Brazil, OR = 0.59, 95% CI: 0.47–0.76, *I^2^* = 76%). However, the six datasets from Asia had a lower OR, and demonstrated less heterogeneity (OR = 0.43, 95% CI: 0.34–0.54, *I^2^* = 51%).

The sensitivity of this analysis was tested by removing all ten datasets contributed by Farooq et al. (1966) [53]. This did not lead to a great change in the findings; in this case the overall OR was 0.52 (95% CI: 0.44–0.61) and *I^2^* remained high at 79%.

### Sanitation and *S. mansoni*

#### Results of individual studies and synthesis of results

**Figure 3:**
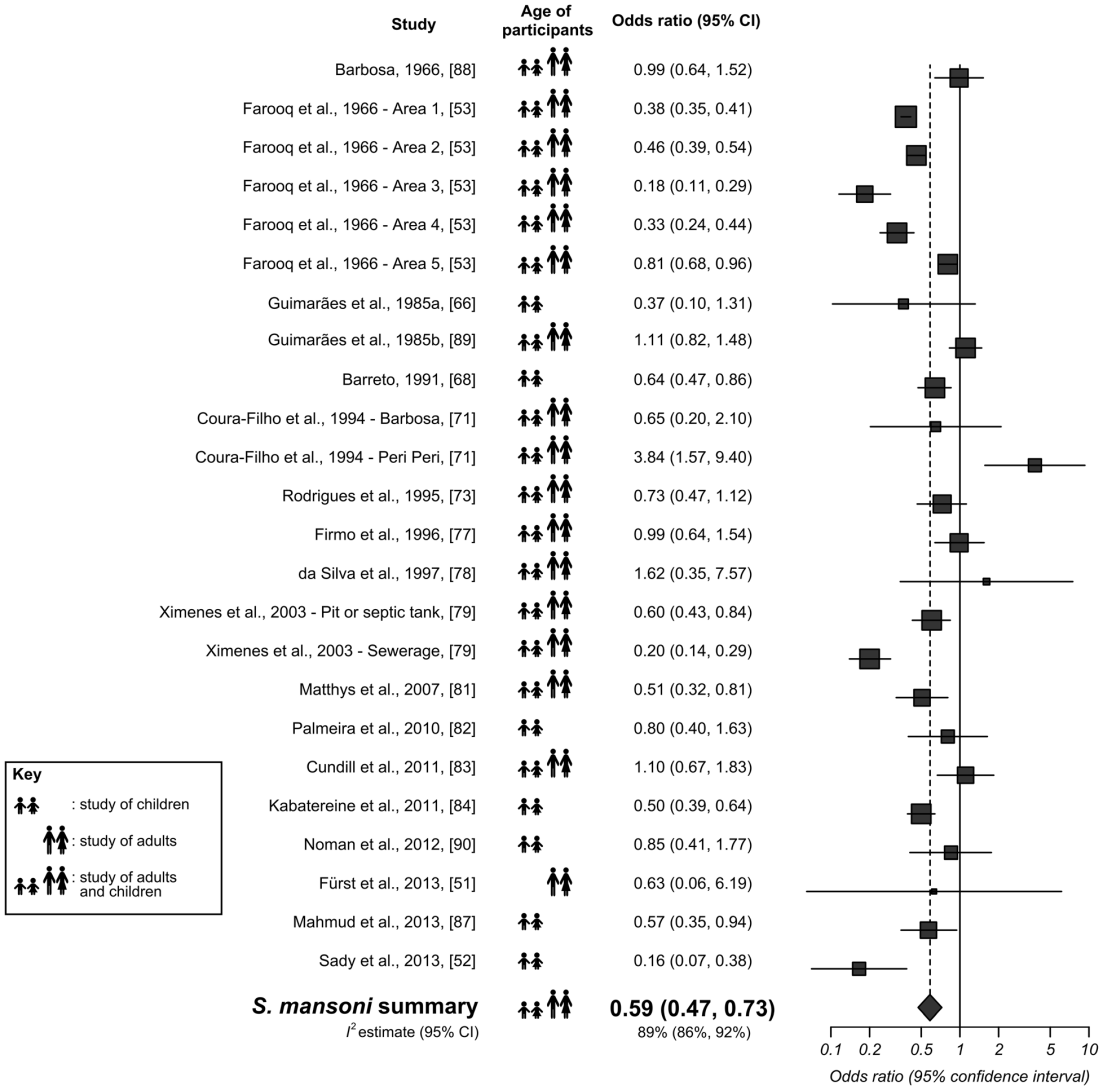
Forest plot for *S. mansoni* infection according to access to adequate sanitation. The square sizes represent the weight given to each dataset, and the black horizontal lines represent 95% confidence intervals. The rhombus is centred on the combined effect size, and its width represents the 95% confidence interval. The *I^2^* estimate is presented beneath the combined effect size.

Overall, adequate sanitation was found to be associated with significantly lower *S. mansoni* infection (OR = 0.59, 95% CI: 0.47–0.73). These studies are summarized and their quality assessment scores presented in Table S3, and their individual and overall combined effect sizes are shown in Figure 3, in which the symbol next each study's effect size denotes whether the participants were children (<18 years of age or in school), adults or a combination. Of the 24 datasets (in 18 studies) reporting *S. mansoni* infection rates according to access to adequate sanitation, 12 reported significantly lower infection rates among those with adequate sanitation. A further 11 datasets found no significant difference, while one dataset found adequate sanitation to be associated with a significantly higher odds of infection.

#### Risk of bias, sub-group analysis, and sensitivity analysis

The funnel plot for sanitation and *S. mansoni* was roughly symmetrical (see Figure S2), although Egger's test revealed a *P* value of 0.10, suggesting that publication bias is possible. Higgins' *I^2^* demonstrated heterogeneity in these datasets, with a value of 89% (95% CI: 86–92%). The following sub-analyses were conducted to determine whether there are differences in age of participants, type of sanitation, or geography that could account for any of this heterogeneity.

Studies of children (seven datasets, OR = 0.54, 95% CI: 0.41–0.72, *I^2^* = 51%), and studies including both adults and children (16 datasets, OR = 0.61, 95% CI: 0.47–0.81, *I^2^* = 92%) both showed similar effect sizes to the overall sanitation and *S. mansoni* meta-analysis. Only one study [51] compared sanitation with *S. mansoni* infection in adults; this had an OR of 0.63 (95% CI: 0.06–6.19).

The 12 datasets comparing *S. mansoni* with ‘latrine’ had an overall OR of 0.54 (95% CI: 0.42–0.71, *I^2^* = 92%). Six datasets compared *S. mansoni* infection with ‘latrine’ or ‘flush toilet’; these had an overall OR of 0.58 (95% CI: 0.41–0.82, *I^2^* = 53%). Two more datasets compared infection with ‘septic tank’ or ‘cesspool’. Their overall OR was 0.77 (95% CI: 0.51–1.17, *I^2^* not calculable). Another two datasets considered ‘sewerage’, and their overall OR was 0.44 (95% CI: 0.09–2.12, *I^2^* not calculable). The remaining two datasets compared *S. mansoni* infection with ‘sewer’ or ‘latrine’. These had a combined OR of 1.65 (95% CI: 0.29–9.37, *I^2^* not calculable).

The 12 datasets from South America (all Brazil) had an overall OR of 0.79 (95% CI: 0.54–1.15, *I^2^* = 86%). Ten more datasets were from Africa, and had an overall OR was 0.46 (95% CI: 0.36–0.59, *I^2^* = 89%). The remaining two studies were carried out in Asia (both in Yemen) – these had an overall OR of 0.38 (95% CI: 0.08–1.90, *I^2^* not calculable).

In the sensitivity analysis, the impact of removing the five datasets contributed by Farooq et al. (1966) [53] was investigated. The OR increased to 0.68 (95% CI: 0.52–0.89, *I^2^* = 81%).

### Sanitation and *S. haematobium*

#### Results of individual studies and synthesis of results

**Figure 4:**
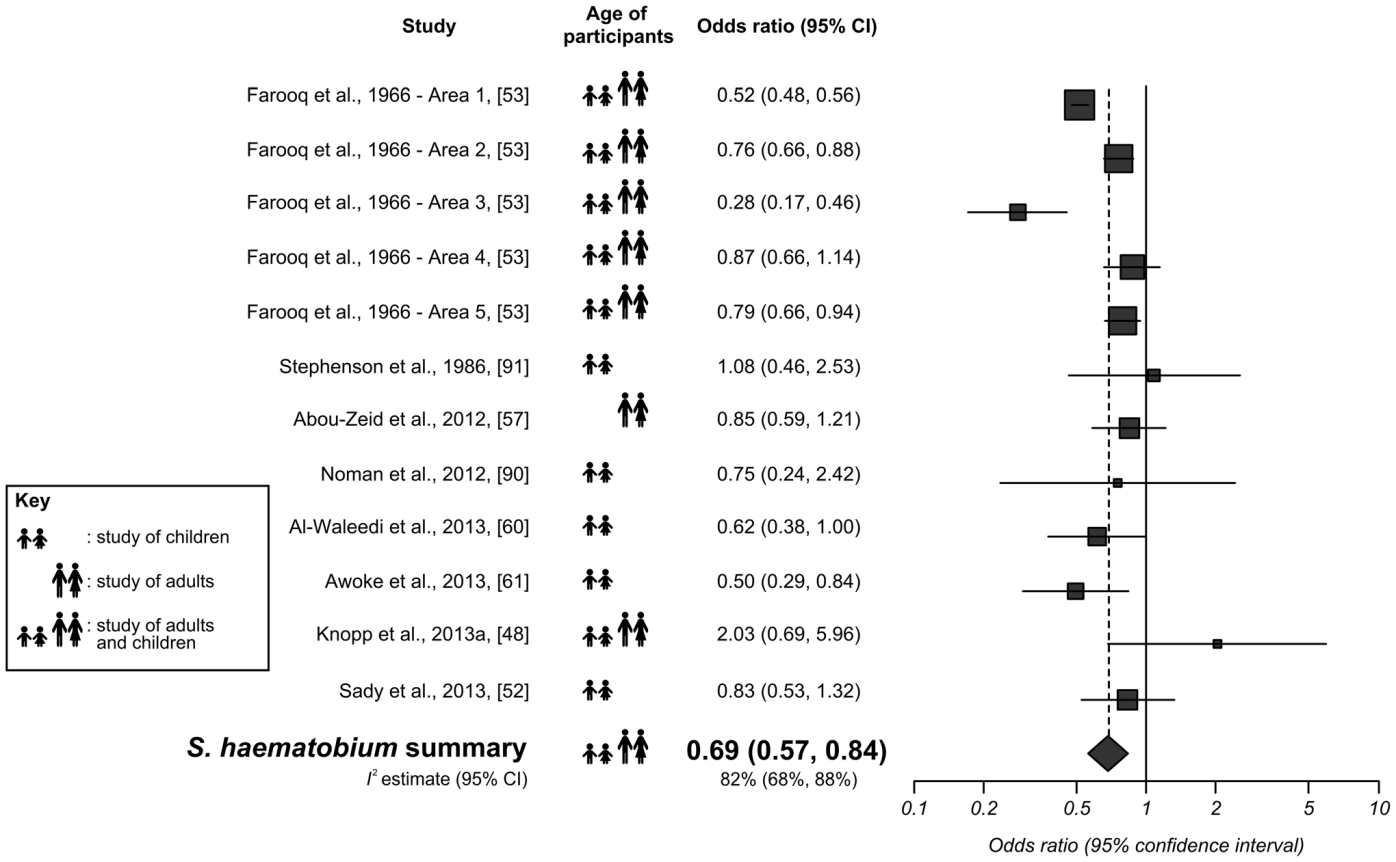
Forest plot for *S. haematobium* infection according to access to adequate sanitation. The square sizes represent the weight given to each dataset, and the black horizontal lines represent 95% confidence intervals. The rhombus is centred on the combined effect size, and its width represents the 95% confidence interval. The *I^2^* estimate is presented beneath the combined effect size.

Sanitation was associated with a significantly lower odds of *S. haematobium* infection, with an OR of 0.69 (95% CI: 0.57–0.84). Eight studies containing 12 datasets comparing *S. haematobium* infection with sanitation were included in this analysis. These are summarized, and their quality assessment scores presented in Table S4. Their individual and combined effect sizes are shown in Figure 4, in which the symbol next each study's effect size denotes whether the participants were children (<18 years of age or in school), adults, or a combination. Five datasets reported a significantly lower odds of *S. haematobium* infection among those with adequate sanitation, and none of the remaining seven showed a significant difference in odds of infection.

#### Risk of bias, sub-group analysis, and sensitivity analysis

The funnel plot for this analysis was roughly symmetrical (see Figure S3), and Egger's test returned a *P* value of 0.21, suggesting that publication bias is unlikely. Higgins' *I^2^* revealed high heterogeneity with a value of 82% (95% CI: 68–88%), and the following sub-analyses were used to explore this heterogeneity.

Studies of children (five datasets, OR = 0.69, 95% CI: 0.53–0.89, *I^2^* = 0%), and studies including both adults and children (six datasets, OR = 0.67, 95% CI: 0.51–0.88, *I^2^* = 91%) both showed similar effect sizes to the overall sanitation and *S. haematobium* meta-analysis. Only one study [57] compared sanitation with *S. haematobium* infection in adults; this had an OR of 0.85 (95% CI: 0.59–1.21).

Eight studies compared *S. haematobium* infection with ‘latrine’ access. These had an overall OR of 0.71 (95% CI: 0.55–0.90, *I^2^* = 88%). Two more studies compared infection with ‘latrine’ or ‘flush toilet’. These had a combined OR of 0.82 (95% CI: 0.54–1.26, *I^2^* not calculable). The remaining two studies defined sanitation as ‘toilet’ [61] and ‘septic tank or cesspool’ [60]. These had ORs of 0.50 (95% CI: 0.29–0.84) and 0.62 (95% CI: 0.38–1.00), respectively. The nine datasets from Africa had a combined OR of 0.68 (95% CI: 0.54–0.86, *I^2^* = 86%), and the three from Asia (all Yemen) had a much lower value of *I^2^* (OR = 0.73, 95% CI: 0.53–1.00, *I^2^* = 0%).

In the sensitivity analysis, the five datasets from Farooq et al. (1966) [53] were removed. This reduced *I^2^* to 21% and, although sanitation was still associated with significantly less infection (OR = 0.77, 95% CI: 0.60–0.98), the difference was now only just statistically significant.

## Discussion

### Summary of Evidence

This is the first systematic review of the association between WASH and *Schistosoma* infection. Access to safe water supplies were found to be associated with significantly less infection with *S. haematobium*, *S. mansoni*, and *S. japonicum*, while adequate sanitation was found to be associated with significantly less infection with both *S. mansoni* and *S. haematobium*. No observational studies were found assessing the association between good hygiene, defined as the use of soap during water contact, and *Schistosoma* infection.

Since schistosome cercariae are susceptible to water treatment and even to water storage [92]–[95], it is reasonable to assume that piped water should not pose a risk of transmission. Thus the ability of safe water sources to prevent *Schistosoma* infection would depend on how well they prevent dermal contact with schistosome-infested environmental water bodies. Jordan et al. (1975) found that provision of piped water to the household was much more effective than centralized community access in preventing water contact and reducing schistosomiasis transmission [96]. However, we found similar ORs for household access and community access (OR = 0.57, 95% CI: 0.28–1.17 for household access rather than use of environmental water bodies, and OR = 0.60, 95% CI: 0.47–0.76 for community access rather than use of environmental water bodies). We identified two observational studies comparing schistosome infection rates in people household access and community access [97], [98], but again, neither study reported significantly lower infection rates in people with household rather than community water supplies. These studies were not included in the meta-analyses since both household and community water sources were ‘safe’, and thus these studies did not meet our inclusion criteria.

Schistosome eggs are released in the urine and the feces of human hosts, but to sustain transmission an egg must enter freshwater and hatch to release a miracidium, which then infects an intermediate host snail [3]. This infected host snail will later release cercariae, which may infect people coming into contact with the water. Thus sanitation's impact upon schistosome transmission is dependent upon its ability to reduce fecal or urinary contamination of freshwater containing intermediate host snails, rather than contamination of the environment in general. Furthermore, owing to exponential reproduction of the parasite within the intermediate host snail, even small numbers of schistosome eggs entering freshwater may give rise to a disproportionately large risk of infection in people coming into contact with that water [95].

The high heterogeneity throughout the meta-analyses could not be attributed to differences in any one of: the schistosome species, ages of study participants, type of sanitation, location of water source, or geography of study (stratified by continent). Perhaps such heterogeneity could be due to a combination of many setting-specific community, ecological, and occupational factors such as the above, presence of intermediate snail hosts, and reasons for water contact, and the input of miracidia into the water. A recent geographical analysis of national survey and demographic health survey data found absence of piped water to be associated with significantly increased infection with *S. haematobium*, but absence of a toilet facility to be associated with a significantly lower odds of *S. mansoni* infection [99]. These findings perhaps reflect that the aforementioned factors are much stronger predictors of infection than WASH, and for example people without adequate WASH may remain uninfected due to a lack of snail intermediate hosts in the locality. Similarly, some studies in this meta-analysis may have included people with inadequate WASH who were nevertheless not exposed to schistosomiasis due to a lack of intermediate host snails nearby, or people with adequate WASH but nevertheless exposed to schistosomes, for example during activities such as fishing.

The lack of observational studies comparing *Schistosoma* infection in people who do and do not use soap during water contact, perhaps reflects the fact that hygienic practices can be more temporary than access to water or sanitation infrastructure. However, in Ethiopia, Erko et al. (2002) found after distribution of soap bars containing endod, the prevalence of schistosome infection in women dropped significantly [100].

### Limitations

In our view, the biggest limitation of the current meta-analysis is the possibility of socioeconomic confounding. Farooq et al. (1966) found that people without latrines showed a higher prevalence of schistosome infection, but that this difference was no longer apparent if the analysis was carried out separately for the sub-populations living in houses made of mud, or bricks, respectively [53]. The authors concluded that the higher infection rates were due to lower SES, which could be measured by house construction or by access to sanitation, rather than any reduction of schistosomiasis transmission arising due to sanitation. Similarly, in Brazil, Gazzinelli et al. (2006) found significantly higher infection rates in households without either a motorcycle or a car, another indicator of low SES [101]. Safe water supplies are also more prevalent amongst those of higher SES, meaning that possible confounding by SES potentially runs through all the meta-analyses presented here. On the other hand, WASH can depend on environmental and other factors, in addition to SES. An example is provided by Barbosa et al. (2013) [102], who compared two rural Brazilian communities and found better sanitation in the community of lower SES. Unfortunately, very few studies reported data that were stratified according to, or adjusted for, SES.

Most of the studies containing data used in the meta-analysis were multivariable analyses, which analyzed the importance of various risk factors (including absence of water and sanitation) for *Schistosoma* infection. As such, they were not focussed on WASH and often did not precisely define the water and sanitation available to, or used by, participants, or indeed distinguish between availability and use of safe and adequate WASH. Regarding sanitation, it was rare for a study to define where latrines or toilets drained to, and we may have therefore included some studies where the adequate sanitation drained directly into lakes or rivers, facilitating schistosome transmission. Very few studies carried out quality control of the schistosomiasis diagnosis (e.g., reading a random sample of 10% of Kato-Katz thick smears by a senior laboratory technician), and none carried out quality control of the WASH data collection (e.g., spot checks whether reported data on availability and use of sanitation are correct).

WASH not being the focus of most studies also raises the possibility of a weak publication bias (the funnel plots and Egger's tests suggested that this was unlikely, but not impossible), and it has also led to imprecisely defined WASH (particularly in the case of those lacking the safe water or adequate sanitation of interest). WASH was always assessed through questionnaires rather than direct inspection. Furthermore, the included studies always compared WASH directly with schistosome infection rates. New research of the relationship between WASH, human exposure through water contact, human contamination of freshwater, cercarial, miracidial and snail populations, and infection rates is needed, in order to provide a deeper understanding of the relationship between WASH and the transmission likelihood of schistosomes. Very few studies reported WASH in a way that allowed for comparison with the JMP definitions [12]. This observation is explained by the fact that the JMP definitions were first put forward only in 2000 [103], and have been further developed subsequently. Many of our included studies were conducted before this. Furthermore, people's use of different water supplies and sanitation may vary with activities and season [91], and therefore the dichotomisation of water supplies into ‘safe’ and ‘unsafe’, and of sanitation into ‘adequate’ and ‘inadequate’ risks oversimplifying access to WASH. (Box 2).

**Box 2. Recommendations for Future Studies Comparing WASH with Schistosome Infection**

Whenever feasible, future studies comparing WASH with schistosome infection rates should (i) have diagnostic quality control performed by a senior laboratory technician; (ii) account for the SES (perhaps through the presence of household assets such as televisions or motorcycles), and schistosomiasis-related knowledge of study participants; (iii) define access to water and sanitation according to the UNICEF and WHO JMP definitions; (iv) assess WASH through inspections rather than questionnaires; (v) consider the presence of additional WASH infrastructure such as sinks and showers in addition to boreholes and latrines; (vi) account for the main reasons for water contact, and the locations of local transmission foci, along with the numbers of snails, miracidia, and cercariae in the water; and (vii) reduce publication bias by presenting results on all risk factors examined, rather than only those significantly associated with infection. Of note, many of the factors highlighted here are also relevant for other NTDs.

Data on infection with different intestinal parasites was often aggregated, with WASH variables presented as risk factors for infection with any parasite. In many cases E-mail addresses were not available, or we received no replies. We were therefore unable to include these studies, despite the fact that the authors had collected data that would qualify for inclusion.

Water contact and thus schistosome transmission, typically takes place outside the home (public exposure), not within the household (domestic exposure) [104], [105]. The individual is exposed to cercariae released by snails infected not just by him- or herself but also by his or her neighbors. With this in mind, one may expect the associations between water, and particularly sanitation, to be most strongly associated with schistosome infection at the community- rather than the household-level, as has been suggested for other diseases [106]. However, very few such analyses have compared schistosome infection rates between communities with different levels of WASH. Yang et al. (2009) did adopt such an approach and found *S. japonicum* infection rates to be significantly lower in communities where more than 50% of people used ‘hygienic lavatories’ [63].

## Conclusions

A meta-analysis of observational studies found both safe water supplies and adequate sanitation to be associated with significantly lower odds of *Schistosoma* infection. This meta-analysis lends support to more consideration of environmental factors and living conditions in schistosomiasis control, and adds to the growing body of evidence about the relationship between WASH and NTDs. Previous meta-analyses have found significant associations between sanitation and STH infection [37], WASH and STH infection [39], and WASH and trachoma [38]. However, the possible confounding caused by factors such as SES shows that adequately powered cluster randomized controlled trials assessing the impact of WASH on human behavior and schistosome infections, and cercarial, miracidial and snail populations, must play an integral role in informing future policy-making. Such studies are needed to inform the potentially crucial role that WASH could play in the elimination of schistosomiasis, in line with World Health Assembly resolution 65.19.

## Supporting Information

Figure S1 **Funnel plot for the safe water and** ***Schistosoma*** **infection meta-analysis.**
(TIF)

Figure S2 **Funnel plot for the adequate sanitation and** ***S. mansoni*** **meta-analysis.**
(TIF)

Figure S3 **Funnel plot for the adequate sanitation and** ***S. haematobium*** **meta-analysis.**
(TIF)

Table S1 **Full list of excluded and included studies.**
(XLSX)

Table S2 **Included study characteristics for the safe water and** ***Schistosoma*** **infection meta-analysis.**
(DOCX)

Table S3 **Included study characteristics for the adequate sanitation and** ***S. mansoni*** **meta-analysis.**
(DOCX)

Table S4 **Included study characteristics for the adequate sanitation and** ***S. haematobium*** **meta-analysis.**
(DOCX)

Text S1 **Study protocol.**
(DOCX)

Text S2 **MOOSE checklist.**
(DOC)

Text S3 **PRISMA checklist.**
(DOC)

Alternative Language Abstract S1 **Helfen sauberes Trinkwasser und sanitäre Einrichtungen gegen Bilharziose?** Systematische Meta-Analyse und Literatur-Review - Translation of abstract into German by David Croll.
(DOCX)
