# Supplementary material for: The Relationship between Water, Sanitation and Schistosomiasis: A Systematic Review and Meta-analysis
Source: PLoS Negl Trop Dis. 2014 Dec 4;8(12):e3296. doi: 10.1371/journal.pntd.0003296 (PMC4256273; doi:10.1371/journal.pntd.0003296)
Supplement: Table S2 — Included study characteristics for the safe water and Schistosoma infection meta-analysis. (DOCX) [file pntd.0003296.s005.docx]

### Included Study Characteristics for the Safe Water and *Schistosoma* Infection Meta-analysis

| **Reference** | **Study design, setting** | **Study population *(selection)*** | **Study quality assessment^†^** | **‘Safe’ water definition** | **‘Unsafe’ water definition** | **Data obtained** | **Dataset** | **Sub-analyses categories**  *(species*  *continent,*  *location of water source)* | **Odds of infection in those with ‘safe water’**  *(number infected with safe water/number uninfected with safe water)* | **Odds of infection in those without ‘safe water’**  *(number infected without safe water/number uninfected without safe water)* | **OR (CI)** |
| --- | --- | --- | --- | --- | --- | --- | --- | --- | --- | --- | --- |
| Abou-Zeid et al., 2012 [[1](#_ENREF_1)] | Descriptive survey in adults in South Kordofan state, Sudan | 1826 adults  *(households randomly selected and all people above 18 years of age and in those households were eligible for inclusion)* | **Diagnostics:** +½  **Number of samples:** +^1^/_3_  **WASH assessment:** 0  **WASH definitions:** 0  **Confounding assessment:** 0  **Reponse rates:** +1  **Other:** 0  **Total:** +1^5^/_6_ | Source of water: closed (pipe/pump) | Source of water: open | 2x2 table | - | *S. haematobium*  Adults  Africa  Home or community vs environmental | 59/946 | 67/754 | 0·70 (0·49-1·01) |
| Al-Shibani et al., 2007 [[2](#_ENREF_2)] | Descriptive survey in schoolchildren in 2 endemic villages in Taiz governorate, Yemen | 152  *(schoolchildren from grades 1, 2, 5 & 6)* | **Diagnostics:** +½  **Number of samples:** +^1^/_3_  **WASH assessment:** 0  **WASH definitions:** 0  **Confounding assessment:** 0  **Reponse rates:** 0  **Other:** 0  **Total:** +^5^/_6_ | Not using pond water | Using pond water | 2x2 table | - | *S. mansoni*  Children  Asia  Home or community vs environmental | 16/40 | 52/44 | 0·34 (0·17-0·69) |
| Al-Waleedi et al., 2013 [[3](#_ENREF_3)] | Descriptive survey in schoolchildren in Abyan governorate, Yemen | 696  *(all schoolchildren in the village)* | **Diagnostics:** +½  **Number of samples:** 0  **WASH assessment:** 0  **WASH definitions:** 0  **Confounding assessment:** 0  **Reponse rates:** +1  **Other:** 0  **Total:** +1½ | Tap water present | Tap water absent | 2x2 table | - | *S. haematobium*  Children  Asia  Home vs undefined | 101/488 | 25/82 | 0·68 (0·41-1·12) |
| Alembrhan et al., 2013 [[4](#_ENREF_4)] | Descriptive survey in schoolchildren in Mekelle, Ethiopia | 457  *(schoolchildren randomly sampled from 4 schools selected purposively)* | **Diagnostics:** +½  **Number of samples:** 0  **WASH assessment:** 0  **WASH definitions:** 0  **Confounding assessment:** 0  **Reponse rates:** +1  **Other:** 0  **Total:** +1½ | Using piped water | Using river water | Adjusted OR from multiple logistic regression | - | *S. mansoni*  Children  Africa  Home or community vs environmental | - | - | 0·37 (0·16-0·86) |
| Arndt et al., 2013 [[5](#_ENREF_5)] | Descriptive survey in HIV-positive adults in Kisii, Kisumu and Kalifi, Kenya | 153  *(aged 18 years and above, selection also based on HIV status)* | **Diagnostics:** +1  **Number of samples:** 0  **WASH assessment:** 0  **WASH definitions:** 0  **Confounding assessment:** 0  **Reponse rates:** +1  **Other:** 0  **Total:** +2 | Water source: piped to house, well ouside of house, or communal water source | Water source: environmental | 2x2 data supplied by authors | - | *S. mansoni*  Adults  Africa  Home or community vs environmental | 5/131 | 1/16 | 0·61 (0·07-5·56) |
| Awoke et al., 2013 [[6](#_ENREF_6)] | Descriptive survey in purposeively sampled schools in Amibera district, Ethiopia | 828  *(randomly selected from the purposively sampled schools)* | **Diagnostics:** +1  **Number of samples:** +^2^/_3_  **WASH assessment:** 0  **WASH definitions:** 0  **Confounding assessment:** 0  **Reponse rates:** +1  **Other:** 0  **Total:** +2^2^/_3_ | Water source: pipe or protected well | Water source: drainage | 2x2 table | - | *S. haematobium*  Children  Africa  Home or community vs environmental | 36/671 | 25/96 | 0·21 (0·12-0·36) |
| Balen et al., 2011 [[7](#_ENREF_7)] | Descriptive survey in 2 villages in Dongting Lake area, Hunan, China | 1298  *(all individuals in both villages were eligible to participate)* | **Diagnostics:** +½  **Number of samples:** +1  **WASH assessment:** 0  **WASH definitions:** 0  **Confounding assessment:** 0  **Reponse rates:** 0  **Other:** 0  **Total:** +1½ | Water source: tap | Water source: lake | Adjusted OR | - | *S. japonicum*  Adults and children  Asia  Home or community vs environmental | - | - | 0·41 (0·30-0·50) |
| Barreto, 1991 [[8](#_ENREF_8)] | Descriptive survey in Santo Antonio de Jesus, Brazil | 1497*  *(all children aged between 12 and 15 years were eligible for inclusion)* | **Diagnostics:** +½  **Number of samples:** +^1^/_3_  **WASH assessment:** 0  **WASH definitions:** 0  **Confounding assessment:** 0  **Reponse rates:** +1  **Other:** 0  **Total:** +1^5^/_6_ | Household has piped water (with or without wastewater drainage) | Water is taken from ponds/open bodies of water | 2x2 table | - | *S. mansoni*  Children  South America  Home vs environmental | 405/982 | 43/67 | 0·64 (0·43-0·96) |
| Coura-Filho et al., 1994 [[9](#_ENREF_9)] | Descriptive surveys in Peri-Peri, Brazil | 337  *(everyone in the study areas was eligible for inclusion, except long-term hospital patients, children below 1 year of age, and the disabled)* | **Diagnostics:** +½  **Number of samples:** +^1^/_3_  **WASH assessment:** 0  **WASH definitions:** 0  **Confounding assessment:** 0  **Reponse rates:** 0  **Other:** 0  **Total:** +^5^/_6_ | ‘Drinking water in the home’ | No ‘drinking water in the home’ | 2x2 table | Barbosa | *S. mansoni*  Adults and children  South America  Home vs undefined | 0.5/0.5 | 40.5/103.5 | 2·56 (0·05-130·97) |
|  |  |  |  |  |  |  | Peri-Peri | *S. mansoni*  Adults and children  South America  Home vs undefined | 47/276 | 7/7 | 0·17 (0·06-0·51) |
| Coura-Filho et al., 1996 [[10](#_ENREF_10)] | Baseline survey of factors associated with schistosomiasis infection in Ravena, Brazil | 998  *(all residents eligible for inclusion)* | **Diagnostics:** +1/2  **Number of samples:** +1/3  **WASH assessment:** 0  **WASH definitions:** 0  **Confounding assessment:** 0  **Reponse rates:** 0  **Other:** 0  **Total:** +^5^/_6_ | Tap water at home | No tap water at home | OR from multivariate analysis | -- | *S. mansoni*  Adults and children  South America  Home vs undefined | - | - | 0·40 (0·16-0·80) |
| Cundill et al., 2011 [[11](#_ENREF_11)] | Baseline of longitudinal study of factors associated with reinfection with *S. mansoni* in Americaninhas, Brazil | 588*  *(aged over 5 years, included in the baseline survey, and met other inclusion criteria)* | **Diagnostics:** +½  **Number of samples:** +1  **WASH assessment:** 0  **WASH definitions:** 0  **Confounding assessment:** +1  **Reponse rates:** 0  **Other:** 0  **Total:** +2½ | Water supply: ‘tap/pipe’ | Water supply: ‘stream, river, lake, dam or other’ | 2x2 table | - | *S. mansoni*  Adults and children  South America  Home or community vs environmental | 15/100 | 54/419 | 1·16 (0·63-2·15) |
| Dame et al., 2006 [[12](#_ENREF_12)] | Descriptive survey in school children in Kaduna state, Nigeria | 306  *(randomly sampled from the 3 schools in the area)* | **Diagnostics:** +1  **Number of samples:** 0  **WASH assessment:** 0  **WASH definitions:** 0  **Confounding assessment:** 0  **Reponse rates:** +1  **Other:** 0  **Total:** +2 | Source of water: tap or well | Source of water: river or pond | 2x2 table | *S. haematobium* | *S. haematobium*  Children  Africa  Home or community vs environmental | 4/212 | 16/74 | 0·09 (0·03-0·27) |
|  |  |  |  |  |  |  | *S. mansoni* | *S. mansoni*  Children  Africa  Home or community vs environmental | 9/207 | 29/61 | 0·09 (0·04-0·20) |
| da Silva et al., 1997 [[13](#_ENREF_13)] | Descriptive survey in Serrano, Cururupu, Brazil | 194 *(systematically sampled from the populaton of Serrano)* | **Diagnostics:** +½  **Number of samples:** 0  **WASH assessment:** 0  **WASH definitions:** 0  **Confounding assessment:** 0  **Reponse rates:** 0  **Other:** 0  **Total:** +½ | Water supply: piped | Water supply: well | 2x2 table | - | *S. mansoni*  Adults and children  South America  Home vs environmental | 59/88 | 12/35 | 1·96 (0·94-4·07) |
| Dawet et al., 2012 [[14](#_ENREF_14)] | Descriptive survey in Plateau state, Nigeria | 242  *(not specified)* | **Diagnostics:** +½  **Number of samples:** 0  **WASH assessment:** 0  **WASH definitions:** 0  **Confounding assessment:** 0  **Reponse rates:** 0  **Other:** 0  **Total:** +½ | Source of water: borehole, tap, well | Source of water: river or stream | 2x2 table | - | *S. haematobium* Adults and children  Africa  Home or community vs environmental | 1/175 | 4/62 | 0·09 (0·01-0·81) |
| de Lima e Costa et al., 1987 [[15](#_ENREF_15)] | Cross-sectional study in Comercinho, Brazil | 1064*  *(all people aged 2 years and above were eligible for inclusion)* | **Diagnostics**: +1  **Number of samples:** +^1^/_3_  **WASH assessment:** 0  **WASH definitions:** 0  **Confounding assessment:** 0  **Reponse rates:** +1  **Other:** 0  **Total:** +2^1^/_3_ | Water supply is piped | Water supply is not piped | 2x2 table | - | *S. mansoni*  Adults and children  South America  Home vs undefined | 285/72 | 629/78 | 0·49 (0·35-0·70) |
| de Lima e Costa et al., 1991 [[16](#_ENREF_16)] | Descriptive survey in Divino, Brazil | 506  *(all inhabitants of the village aged over 1 year were eligible for inclusion)* | **Diagnostics:** +½  **Number of samples:** +^1^/_3_  **WASH assessment:** 0  **WASH definitions:** 0  **Confounding assessment:** 0  **Reponse rates:** +1  **Other:** 0  **Total:** +1^5^/_6_ | Water suply is piped | Water supply is not piped (includes wells) | 2x2 table | - | *S. mansoni*  Adults and children  South America  Home vs undefined | 55/108 | 143/200 | 0·71 (0·48-1·05) |
| de Lima e Costa et al., 1994 [[17](#_ENREF_17)] | Descriptive study in Comercinho, Minas Gerais, Brazil | 1162  *(inhabitants aged 2 years and above were eligible for inclusion)* | **Diagnostics:** +½  **Number of samples:** +^1^/_3_  **WASH assessment:** 0  **WASH definitions:** +1  **Confounding assessment:** 0  **Reponse rates:** +1  **Other:** 0  **Total:** +2^5^/_6_ | Piped water in the home | No piped water in the home | 2x2 table | - | *S. mansoni*  Adults and children  South America  Home vs undefined | 417/198 | 460/87 | 0·40 (0·30-0·53) |
| Farooq et al., 1966 [[18](#_ENREF_18)] | Descriptive survey in the Egypt-49 project area, Egypt | 23,888  *(random sample of individuals living in the areas)* | **Diagnostics:** +1  **Number of samples:** +^1^/_3_  **WASH assessment:** 0  **WASH definitions:** 0  **Confounding assessment:** +1  **Reponse rates:** +1  **Other:** 0  **Total:** +3^1^/_3_ | Standpipe | Canal water | 2x2 table | *S. haematobium* - Area 1 - project area | *S. haematobium*  Adults and children  Africa  Community vs environmental | 2606/7860 | 429/819 | 0·63 (0·56-0·72) |
|  |  |  |  |  |  |  | *S. haematobium* - Area 2 - rural division | *S. haematobium*  Adults and children  Africa  Community vs environmental | 925/2450 | 224/481 | 0·81 (0·68-0·97) |
|  |  |  |  |  |  |  | *S. haematobium* - Area 3 - urban division | *S. haematobium*  Adults and children  Africa  Community vs environmental | 451/3103 | 5/21 | 0·61 (0·23-1·63) |
|  |  |  |  |  |  |  | *S. haematobium* - Area 4 - reclamation division | *S. haematobium*  Adults and children  Africa  Community vs environmental | 475/1124 | 62/144 | 0·98 (0·72-1·35) |
|  |  |  |  |  |  |  | *S. haematobium* - Area 5 - control division | *S. haematobium*  Adults and children  Africa  Community vs environmental | 758/1180 | 138/173 | 0·81 (0·63-1·03) |
|  |  |  |  |  |  |  | *S. mansoni* - Area 1 - project area | *S. mansoni*  Adults and children  Africa  Community vs environmental | 2166/8300 | 508/740 | 0·38 (0·34-0·43) |
|  |  |  |  |  |  |  | *S. mansoni* - Area 2 - rural division | *S. mansoni*  Adults and children  Africa  Community vs environmental | 635/2741 | 254/451 | 0·41 (0·34-0·49) |
|  |  |  |  |  |  |  | *S. mansoni* - Area 3 - urban division | *S. mansoni*  Adults and children  Africa  Community vs environmental | 490/3064 | 4/22 | 0·88 (0·3-2·56) |
|  |  |  |  |  |  |  | *S. mansoni* - Area 4 - reclamation division | *S. mansoni*  Adults and children  Africa  Community vs environmental | 190/1409 | 69/137 | 0·27 (0·19-0·37) |
|  |  |  |  |  |  |  | *S. mansoni* - Area 5 - control division | *S. mansoni*  Adults and children  Africa  Community vs environmental | 847/1091 | 181/130 | 0·56 (0·44-0·71) |
| Fentie et al., 2013 [[19](#_ENREF_19)] | Descriptive survey in schoolchildren in Lake Tana Basin, Ethiopia | 520  *(stratified sampling according to school, age and gender)* | **Diagnostics:** +1  **Number of samples:** 0  **WASH assessment:** 0  **WASH definitions:** 0  **Confounding assessment:** +1  **Reponse rates:** +1  **Other:** 0  **Total:** +3 | Not unsafe drinking water sources | Unsafe drinking water sources | Odds ratio from bivariate analysis | *-* | *S. mansoni*  Children  Africa  Home or community vs environmental | - | - | 0·52 (0·33-0·84) |
| Firmo et al., 1996 [[20](#_ENREF_20)] | Case-control survey in Gorduras, Minas Gerais, Brazil | 916*  *(households selected randomly and all residents in these households were eligible for inclusion)* | **Diagnostics:** +½  **Number of samples:** +^2^/_3_  **WASH assessment:** 0  **WASH definitions:** +1  **Confounding assessment:** 0  **Reponse rates:** +1  **Other:** 0  **Total:** +2^5^/_6_ | Piped water in the household | No piped water in the household | 2x2 table | - | *S. mansoni*  Adults and children  South America  Home vs undefined | 399/425 | 52/40 | 0·72 (0·47-1·11) |
| Fürst et al., 2013 [[21](#_ENREF_21)] | Descriptive survey as part of the Taabo health demographic surveillance system, in south-central Côte d'Ivoire | 195  *(adults in a stratified random sample of approximately 7% of households in the area of the Taabo health demographic surveillance system)* | **Diagnostics:** +½  **Number of samples:** +^1^/_3_  **WASH assessment:** 0  **WASH definitions:** +1  **Confounding assessment:** 0  **Reponse rates:** 0  **Other:** 0  **Total:** +1^5^/_6_ | No use of natural water bodies as a drinking water source | Use of natural water bodies as a drinking water source | 2x2 table supplied by the authors | - | *S. mansoni*  Adults  Africa  Home or community vs environmental | 2/121 | 2/70 | 0·58 (0·08-4·20) |
| Guimarães et al., 1985b [[22](#_ENREF_22)] | Descriptive survey of schoolchildren in Ilha, Brazil | 167*  *(all children at the community’s school were eligible for inclusion)* | **Diagnostics:** +1  **Number of samples:** +^1^/_3_  **WASH assessment:** 0  **WASH definitions:** 0  **Confounding assessment:** 0  **Reponse rates:** +1  **Other:** 0  **Total:** +2^1^/_3_ | Piped water to the household | No piped water to the household | 2x2 table | - | *S. mansoni*  Children  South America  Home vs undefined | 27/49 | 41/50 | 0·67 (0·36-1·26) |
| Howarth et al., 1988 [[23](#_ENREF_23)] | Descriptive survey of secondary schoolchildren Ankilivalo, Madagascar | 133*  *(not specified)* | **Diagnostics**: +1  **Number of samples:** 0  **WASH assessment:** 0  **WASH definitions:** 0  **Confounding assessment:** 0  **Reponse rates:** 0  **Other:** -1  (*Due to time of sample collection)*  **Total:** 0 | Water source: well | Water source: canal | 2x2 table | - | *S. haematobium*  Children  Africa  Community vs environmental | 67/34 | 12/20 | 3.28 (1·44-7·50) |
| Kabatereine et al., 2011 [[24](#_ENREF_24)] | Descriptive survey at Lake Victoria, Uganda | 1784  *(15 children were randomly selected in each village)* | **Diagnostics:** +1  **Number of samples:** +^1^/_3_  **WASH assessment:** 0  **WASH definitions:** 0  **Confounding assessment:** 0  **Reponse rates:** +1  **Other:** 0  **Total:** +2^1^/_3_ | Source of household water: Clean | Source of household water: River or lake | Odds ratio from multivariate stepwise logistic regresion model | - | *S. mansoni*  Children  Africa  Home or community vs environmental | - | - | 0·78 (0·60-1·01) |
| Knopp et al., 2013b [[25](#_ENREF_25)] | Baseline parasitological and risk factor survey in adults in Zanzibar | 3995*  *(adults aged 20-55 years who provided informed consent)* | **Diagnostics:** +½  **Number of samples:** 0  **WASH assessment:** 0  **WASH definitions:** 0  **Confounding assessment:** 0  **Reponse rates:** +1  **Other:** 0  **Total:** +1½ | Not using natural freshwater | Using natural freshwater | 2x2 tables supplied by authors | Unguja | *S. haematobium*  Adults  Africa  Home or community vs environmental | 44/1820 | 13/257 | 0·48 (0·25-0·90) |
|  |  |  |  |  |  |  | Permba | *S. haematobium*  Adults  Africa  Home or community vs environmental | 43/1080 | 59/679 | 0·46 (0·31-0·69) |
| Kubasta, 1964 [[26](#_ENREF_26)] | Survey of schoolchildren in Harar, Ethiopia | 134*  *(schoolboys between ages of 9 and 18 years)* | **Diagnostics:** +1  **Number of samples:** 0  **WASH assessment:** 0  **WASH definitions:** 0  **Confounding assessment:** 0  **Reponse rates:** 0  **Other:** 0  **Total:** +1 | Piped water available at or near home | Piped water not available at or near home | 2x2 table | - | *S. mansoni*  Children  Africa  Home vs undefined | 33/20 | 62/19 | 0·51 (0·24-1·08) |
| Mahmud et al., 2013 [[27](#_ENREF_27)] | Survey of schoolchildren in 12 schools in northern Ethiopia | 300*  *(schoolchildren randomly selected from school rosters)* | **Diagnostics:** +½  **Number of samples:** +^2^/_3_  **WASH assessment:** 0  **WASH definitions:** 0  **Confounding assessment:** 0  **Reponse rates:** +1  **Other:** 0  **Total:** +3^1^/_3_ | Household water source: pipe or hand pump | Household water source: wells and streams | 2x2 table | - | *S. mansoni*  Children  Africa  Home vs undefined | 70/386 | 14/130 | 1·68 (0·92-3·09) |
| Marcal Junior et al., 1993 [[28](#_ENREF_28)] | Matched case-control study in Pedro Toledo, Brazil | 192  *(96 positives and 96 controls matched for sex, age and place of residence)* | **Diagnostics:** +½  **Number of samples:** +^1^/_3_  **WASH assessment:** 0  **WASH definitions:** 0  **Confounding assessment:** 0  **Reponse rates:** +1  **Other:** 0  **Total:** +1^5^/_6_ | Adequate source of drinking water | Inadequate source of drinking water | Odds ratio frombivariate analysis of matched case-control pairs | - | *S. mansoni*  Adults and children  South America  Home or community vs environmental | - | - | 1.00 (0·48-2·10) |
| Matthys et al., 2007 [[29](#_ENREF_29)] | Cross-sectional descriptive survey in Man, Côte d'Ivoire | 716  *(households randomly selected and all people in these households were eligible for inclusion)* | **Diagnostics:** +1  **Number of samples:** +^1^/_3_  **WASH assessment:** 0  **WASH definitions:** 0  **Confounding assessment:** 0  **Reponse rates:** 0  **Other:** 0  **Total:** +1^1^/_3_ | Non-use of water from irrigation wells and ponds | Use of water from irrigation wells and ponds | Odds ratio from multivariate model | - | *S. mansoni*  Adults and children  Africa  Home or community vs environmental | - | - | 0·40 (0·27-0·63) |
| Nworie et al., 2012 [[30](#_ENREF_30)] | Descriptive survey in primary schoolchildren in Ebonyi state, Nigeria | 500  *(schoolchildren aged 5-15 years were randomly selected from five schools)* | **Diagnostics:** +½  **Number of samples:** 0  **WASH assessment:** 0  **WASH definitions:** 0  **Confounding assessment:** 0  **Reponse rates:** +1  **Other:** 0  **Total:** +1½ | Source of water: borehole or well | Source of water: pond or stream | 2x2 table | - | *S. haematobium*  Children  Africa  Community vs environmental | 2/238 | 47/213 | 0·04 (0·01-0·08) |
| Palmeira et al., 2010 [[31](#_ENREF_31)] | Survey of schoolchildren in two municpalities in the state of Alagos, Brazil | 329*  *(all school children aged 7-15 years in the two municipalities studied were eligible for inclusion)* | **Diagnostics:** +½  **Number of samples:** +^2^/_3_  **WASH assessment:** 0  **WASH definitions:** 0  **Confounding assessment:** +1  **Reponse rates:** 0  **Other:** 0  **Total:** +2^1^/_6_ | Water supply: public supply or clandestine connection | Water supply: well | 2x2 table | - | *S. mansoni*  Children  South America  Home or community vs environmental | 48/175 | 12/34 | 0·78 (0·37-1·62) |
| Reuben et al., 2013 [[32](#_ENREF_32)] | Survey of schoolchildren in Lafia, Nigeria | 160  *(schoolchilren randomly selected from eight secondary schools)* | **Diagnostics:** +½  **Number of samples:** 0  **WASH assessment:** 0  **WASH definitions:** 0  **Confounding assessment:** 0  **Reponse rates:** +1  **Other:** 0  **Total:** +1½ | Water source: well, borehole or tap | Water source: river or stream | 2x2 table | - | *S. haematobium*  Children  Africa  Home or community vs environmental | 11/81 | 15/53 | 0·48 (0·21-1·12) |
| Rodrigues et al., 1995 [[33](#_ENREF_33)] | Descriptive survey in Itinga, Brazil | 324*  *(all inhabitants of the village of Ponte Do Pasmado were eligible for inclusion)* | **Diagnostics:** +1  **Number of samples:** +^1^/_3_  **WASH assessment:** 0  **WASH definitions:** 0  **Confounding assessment:** 0  **Reponse rates:** +1  **Other:** 0  **Total:** +2^1^/_3_ | Origin of water: cistern in the dwelling | Origin of water: stream | 2x2 table | - | *S. mansoni*  Adults and children  South America  Home vs environmental | 13/33 | 150/128 | 0·34 (0·17-0·67) |
| Sady et al., 2013 [[34](#_ENREF_34)] | Decsriptive survey in 10 districts in Western Yemen | 400  *(households randomly selected and all children up to 15 years of age were eligible for inclusion in the study)* | **Diagnostics:** +1  **Number of samples:** 0  **WASH assessment:** 0  **WASH definitions:** 0  **Confounding assessment:** 0  **Reponse rates:** 0  **Other:** 0  **Total:** +1 | Source of household water: safe (piped) | Source of household water: unsafe (stream, rain, well, etc) | 2x2 table supplied by authors | *S. haematobium* | *S. haematobium*  Children  Asia  Home or community vs environmental | 18/95 | 77/210 | 0·52 (0·29-0·91) |
|  |  |  |  |  |  |  | *S. mansoni* | *S. mansoni*  Children  Asia  Home or community vs environmental | 8/105 | 29/258 | 0·68 (0·30-1·53) |
| Soares et al., 1995 [[35](#_ENREF_35)] | Descriptive study in Paracambi, Brazil | 1196  *(all households in Paracambi eligible for inclusion)* | **Diagnostics:** +½  **Number of samples:** 0  **WASH assessment:** 0  **WASH definitions:** 0  **Confounding assessment:** 0  **Reponse rates:** 0  **Other:** 0  **Total:** +½ | Source of residential water supply: treated | Source of residential water supply: spring, well or stream | 2x2 table | - | *S. mansoni*  Adults and children  South America  Home or community vs environmental | 8/478 | 24/686 | 0·48 (0·21-1·07) |
| Umar and Parakoyi, 2005 [[36](#_ENREF_36)] | Descriptive survey of schoolchildren at six schools near the Bakalori Dam, Nigeria | 240  *(schools chosen randomly and children selected randomly from grades 4, 5 and 6)* | **Diagnostics:** +½  **Number of samples:** 0  **WASH assessment:** 0  **WASH definitions:** 0  **Confounding assessment:** 0  **Reponse rates:** +1  **Other:** 0  **Total:** +1½ | Source of water: Borehole or well | Source of water: River, dam or canal | 2x2 table | - | *S. haematobium*  Children  Africa  Community vs environmental | 22/46 | 79/93 | 0·56 (0·31-1·02) |
| Watts and El Katsha, 1995 [[37](#_ENREF_37)] | Descriptive survey in the Nile Delta, Egypt | 967  *(random sample of 15% of houses in Kom el Akhdar and 6% of houses in El Roda)* | **Diagnostics:** +½  **Number of samples:** +^1^/_3_  **WASH assessment:** 0  **WASH definitions:** +1  **Confounding assessment:** 0  **Reponse rates:** 0  **Other:** 0  **Total:** +1^5^/_6_ | Household has a water connection | Household does not have a water connection | 2x2 table | El Roda | *S. mansoni*  Adults and children  Africa  Home vs undefined | 31/164 | 81/178 | 0·42 (0·26-0·66) |
|  |  |  |  |  |  |  | Kom el Akhdar | *S. mansoni*  Adults and children  Africa  Home vs undefined | 61/711 | 19/201 | 0·91 (0·53-1·55) |
| Ximenes et al., 2003 [[38](#_ENREF_38)] | Descriptive survey in São Lourenço da Mata, Brazil | 1723 families  *(households randomly selected and all those aged 10-25 years in those households were eligible for inclusion)* | **Diagnostics:** +½  **Number of samples:** 0  **WASH assessment:** 0  **WASH definitions:** 0  **Confounding assessment:** +1  **Reponse rates:** 0  **Other:** 0  **Total:** +1½ | Water supply: piped water inside the house or covered well, public standpipe, piped water but not inside the house | Water supply: stream, river or uncovered well | Odds ratios from biavariate models | Home vs environemntal | *S. mansoni*  Adults and children  South America  Home vs environmental | - | - | 0·78 (0·61-0·99) |
|  |  |  |  |  |  |  | Community vs environmental | *S. mansoni*  Adults and children  South America  Community vs environmental | - | - | 0·30 (0·23-0·39) |
| Yang et al., 2009 [[39](#_ENREF_39)] | Cross-sectional descriptive survey in 16 villages in Hunan, China | 10,108*  *(all residents in these 16 villages, aged 6 years or over, were eligible for inclusion in this study)* | **Diagnostics:** +½  **Number of samples:** +^1^/_3_  **WASH assessment:** 0  **WASH definitions:** 0  **Confounding assessment:** 0  **Reponse rates:** 0  **Other:** 0  **Total:** +^5^/_6_ | Drinking water source: safe | Drinking water source: infested | 2x2 table | - | *S. japonicum*  Adults and children  Asia  Home or community vs environmental | 227/7813 | 167/1901 | 0·33 (0·27-0·41) |

* WASH and schistosomiasis data not available for complete study population. Population reported is the number with WASH and schistosomiasis data available

^†^ Quality of studies was assessed by assigning scores for diagnostic approach (+1 if sedimentation was used for intestinal schistosomiasis or multiple diagnostic approaches used, +1/2 for Kato-Katz or urine filtration, 0 otherwise); number of samples analysed (+1 if multiple stool/urine samples taken, +2/3 if slides checked by another technician, +1/3 if multiple slides read from the same sample, 0 otherwise); WASH assessment (+1 if household visit and inspection, or at least some spot checks, 0 if questionnaire outside of the home or WASH assessment method not defined); WASH definitions (+1 if they allow for comparison with JMP indicators,[[40](#_ENREF_40)] 0 otherwise); confounding assessment (+1 for data being split according to non-WASH variables found to be predictive of infection, 0 otherwise); response rates (+1 for above 80%, 0 for below 80% or not defined); and other (+1 for additional strengths and -1 for additional weaknesses).

## References

1. Abou-Zeid AHA, Abkar TA, Mohamed RO (2012) Schistosomiasis and soil-transmitted helminths among an adult population in a war affected area, Southern Kordofan state, Sudan. Parasit Vectors 5: 133.

2. Al-Shibani LA, El-Heggiagi MB, Burshan NM, Bassiouny HK (2007) Development of schistosomal school-based health education model for Yemeni schoolchildren. J Egypt Soc Parasitol 37: 649-658.

3. Al-Waleedi AA, El-Nimr NA, Hasab AA, Bassiouny HK, Al-Shibani LA (2013) Urinary schistosomiasis among schoolchildren in Yemen: prevalence, risk factors, and the effect of a chemotherapeutic intervention. J Egypt Public Health Assoc 88: 130-136.

4. Alembrhan A, Tadesse D, Zewdneh T (2013) Infection prevalence of *Schistosoma mansoni* and associated risk factors among schoolchildren in suburbs of Mekelle city, Tigray, Northern Ethiopia. MEJS 5: 174-188.

5. Arndt MB, John-Stewart G, Richardson BA, Singa B, Van Lieshout L, et al. (2013) Impact of helminth diagnostic test performance on estimation of risk factors and outcomes in HIV-positive adults. PLoS One 8: e81915.

6. Awoke W, Bedimo M, Tarekegn M (2013) Prevalence of schistosomiasis and associated factors among students attending at elementary schools in Amibera District, Ethiopia. Open J Prev Med 3: 199-204.

7. Balen J, Raso G, Li YS, Zhao ZY, Yuan LP, et al. (2011) Risk factors for helminth infections in a rural and a peri-urban setting of the Dongting Lake area, People’s Republic of China. Int J Parasitol 41: 1165-1173.

8. Barreto ML (1991) Geographical and socioeconomic factors relating to the distribution of *Schistosoma mansoni* infection in an urban area of north-east Brazil. Bull World Health Organ 69: 93-102.

9. Coura-Filho P, Rocha RS, Farah MW, da Silva GC, Katz N (1994) Identification of factors and groups at risk of infection with *Schistosoma mansoni*: a strategy for the implementation of control measures? Rev Inst Med Trop São Paulo 36: 245-253.

10. Coura-Filho P, Rocha RS, Lamartine SS, Farah MW, de Resende DF, et al. (1996) Control of schistosomiasis mansoni in Ravena (Sabara, state of Minas Gerais, Brazil) through water supply and quadrennial treatments. Mem Inst Oswaldo Cruz 91: 659-664.

11. Cundill B, Alexander N, Bethony JM, Diemert D, Pullan RL, et al. (2011) Rates and intensity of re-infection with human helminths after treatment and the influence of individual, household, and environmental factors in a Brazilian community. Parasitology 138: 1406-1416.

12. Dame JG, Banwat EB, Egah DZ, Shabi ME (2006) Schistosomiasis among students in a local government area of Kaduna State in Northern Nigeria. Highland Med Res J 4: 60-69.

13. da Silva AA, Cutrim RN, de Britto e Alves MT, Coimbra LC, Tonial SR, et al. (1997) Water-contact patterns and risk factors for *Schistosoma mansoni* infection in a rural village of northeast Brazil. Rev Inst Med Trop São Paulo 39: 91-96.

14. Dawet A, Benjamin CB, Yakubu DP (2012) Prevalence and intensity of *Schistosoma haematobium* among residents of Gwong and Kabong in Jos north local government area, Plateau State, Nigeria. International Journal of Tropical Medicine 7: 69-73.

15. de Lima e Costa MFF, Magalhães MHA, Rocha RS (1987) Water-contact patterns and socioeconomic variables in the epidemiology of schistosomiasis mansoni in an endemic area in Brazil. Bull World Health Organ 65: 57-66.

16. de Lima e Costa MFF, Rocha RS, Leite MLC, Carneiro RG, Colley D, et al. (1991) A multivariate analysis of socio-demographic factors, water contact patterns and *Schistosoma mansoni* infection in an endemic area in Brazil. Rev Inst Med Trop São Paulo 33: 58-63.

17. de Lima e Costa MFF, Rocha RS, Magalhaes MH, Katz N (1994) [A hierarchical model for analysis of socio-economic variables and water contact patterns associated with the hepatosplenic form of schistosomiasis]. Cad Saúde Pública 10 Suppl 2: 241-253. (in Portuguese)

18. Farooq M, Nielsen J, Samaan SA, Mallah MB, Allam AA (1966) The epidemiology of *Schistosoma haematobium* and *S. mansoni* infections in the Egypt-49 project area. 2. Prevalence of bilharziasis in relation to personal attributes and habits. Bull World Health Organ 35: 293-318.

19. Fentie T, Erqou S, Gedefaw M, Desta A (2013) Epidemiology of human fascioliasis and intestinal parasitosis among schoolchildren in lake Tana Basin, northwest Ethiopia. Trans R Soc Trop Med Hyg 107: 480-486.

20. Firmo JOA, Costa MF, Guerra HL, Rocha RS (1996) Urban schistosomiasis: morbidity, sociodemographic characteristics and water contact patterns predictive of infection. Int J Epidemiol 25: 1292-1300.

21. Fürst T, Ouattara M, Silué KD, N'Goran DN, Adiossan LG, et al. (2013) Scope and limits of an anamnestic questionnaire in a control-induced low-endemicity helminthiasis setting in south-central Côte d'Ivoire. PLoS One 8: e64380.

22. Guimarães MD, Costa MF, de Lima LB, Moreira MA (1985) [Clinico-epidemiological study of schistosomiasis mansoni in school children of Ilha, municipality of Arcos, MG (Brazil) 1983]. Rev Saude Publica 19: 8-17. (in Portuguese)

23. Howarth SE, Wilson JM, Ranaivoson E, Crook SE, Denning AM, et al. (1988) Worms, wells and water in Western Madagascar. J Trop Med Hyg 91: 255-264.

24. Kabatereine NB, Standley CJ, Sousa-Figueiredo JC, Fleming FM, Stothard JR, et al. (2011) Integrated prevalence mapping of schistosomiasis, soil-transmitted helminthiasis and malaria in lakeside and island communities in Lake Victoria, Uganda. Parasit Vectors 4: 232.

25. Knopp S, Person B, Ame SM, Mohammed KA, Ali SM, et al. (2013) Elimination of schistosomiasis transmission in Zanzibar: baseline findings before the onset of a randomized intervention trial. PLoS Negl Trop Dis 7: e2474.

26. Kubasta M (1964) Schistosomiasis mansoni in the Harar province. Ethiop Med J 2: 260-271.

27. Mahmud MA, Spigt M, Bezabih AM, Lopez Pavon I, Dinant G-J, et al. (2013) Risk factors for intestinal parasitosis, anaemia, and malnutrition among school children in Ethiopia. Pathog Glob Health 107: 58-65.

28. Marcal Junior O, Hotta LK, Patucci RM, Glasser CM, Dias LC (1993) Schistosomiasis mansoni in an area of low transmission. II. Risk factors for infection. Rev Inst Med Trop São Paulo 35: 331-335.

29. Matthys B, Tschannen AB, Tian-Bi NT, Comoe H, Diabate S, et al. (2007) Risk factors for *Schistosoma mansoni* and hookworm in urban farming communities in western Côte d’Ivoire. Trop Med Int Health 12: 709-723.

30. Nworie O, Nya O, Anyim C, Okoli CS, Okonkwo EC (2012) Prevalence of urinary schistosomiasis among primary school children in Afikpo North Local government area of Ebonyi State. Annals of Biological Research 3: 3894-3897.

31. Palmeira DCC, de Carvalho AG, Rodrigues K, Couto JLA (2010) [Prevalence of *Schistosoma mansoni* infection in two municipalities of the State of Alagoas, Brazil]. Rev Soc Bras Med Trop 43: 313-317. (in Portuguese)

32. Reuben RC, Tanimu H, Musa JA (2013) Epidemiology of urinary schistosomiasis among secondary school students in Lafia, Nasarawa State, Nigeria. Journal of Biology, Agriculture and Healthcare 3: 73-82.

33. Rodrigues RN, Murta C, Teixeira Junior MA, Cury GC, Rocha MO (1995) Clinical-epidemiologic study of schistosomiasis mansoni in Ponte do Pasmado, a village in the municipality of Itinga, state of Minas Gerais, Brazil, 1992. Rev Inst Med Trop São Paulo 37: 81-85.

34. Sady H, Al-Mekhlafi HM, Mahdy MAK, Lim YAL, Mahmud R, et al. (2013) Prevalence and associated factors of schistosomiasis among children in Yemen: implications for an effective control programme. PLoS Negl Trop Dis 7: e2377.

35. Soares MS, Barreto MG, da Silva CL, Pereira JB, Moza PG, et al. (1995) Schistosomiasis in a low prevalence area: incomplete urbanization increasing risk of infection in Paracambi, RJ, Brazil. Mem Inst Oswaldo Cruz 90: 451-458.

36. Umar AS, Parakoyi DB (2005) The prevalence and intensity of urinary schistosomiasis among school children living along the Bakalori Dam, Nigeria. Niger Postgrad Med J 12: 168-172.

37. Watts S, El Katsha S (1995) Changing environmental conditions in the Nile delta: health and policy implications with special reference to schistosomiasis. Int J Environ Heal R 5: 197-212.

38. Ximenes R, Southgate B, Smith PG, Guimaraes Neto L (2003) Socioeconomic determinants of schistosomiasis in an urban area in the Northeast of Brazil. Rev Panam Salud Publica 14: 409-421.

39. Yang JZ, Zhao ZY, Li YS, Krewski D, Wen SW (2009) A multi-level analysis of risk factors for *Schistosoma japonicum* infection in China. Int J Infect Dis 13: E407-E412.

40. WHO, UNICEF (2013) Progress on sanitation and drinking-water - 2013 update. Geneva: World Health Organization.
