# Supplementary material for: The Relationship between Water, Sanitation and Schistosomiasis: A Systematic Review and Meta-analysis
Source: PLoS Negl Trop Dis. 2014 Dec 4;8(12):e3296. doi: 10.1371/journal.pntd.0003296 (PMC4256273; doi:10.1371/journal.pntd.0003296)
Supplement: Table S3 — Included study characteristics for the adequate sanitation and S. mansoni meta-analysis. (DOCX) [file pntd.0003296.s006.docx]

### Included Study Characteristics for the Adequate Sanitation and *S. mansoni* Meta-analysis

| **Reference** | **Study design, setting** | **Study population *(selection)*** | **‘Adequate’ sanitation definition** | **Study quality assessment^†^** | **Data obtained** | **Dataset** | **Sub-analyses categories**  *(type of sanitation,*  *continent)* | **Odds of infection in those with ‘adequate sanitation’**  *(number infected with adequate sanitation /number uninfected with adequate sanitation)* | **Odds of infection in those without ‘adequate sanitation’**  *(number infected with adequate sanitation /number uninfected without improved sanitation)* | **OR (CI)** |
| --- | --- | --- | --- | --- | --- | --- | --- | --- | --- | --- |
| Barbosa, 1966 [[1](#_ENREF_1)] | Descriptive survey in Agua Preta, Brazil | 955*  *(people of any age randomly sampled from the village)* | Latrine | **Diagnostics:** +1  **Number of samples:** 0  **WASH assessment:** 0  **WASH definitions:** 0  **Confounding assessment:** 0  **Reponse rates:** 0  **Other:** 0  **Total:** +1 | 2x2 table | - | Latrine  Adults and children  South America | 492/54 | 369/40 | 0·99 (0·64-1·52) |
| Barreto, 1991 [[2](#_ENREF_2)] | Descriptive survey in Santo Antonio de Jesus, Brazil | 1494*  *(all children aged between 12 and 15 years were eligible for inclusion)* | Latrine or flush toilet | **Diagnostics:** +½  **Number of samples:** +^1^/_3_  **WASH assessment:** 0  **WASH definitions:** 0  **Confounding assessment:** 0  **Reponse rates:** +1  **Other:** 0  **Total:** +1^5^/_6_ | 2x2 table | - | Latrine or flush toilet  Children  South America | 359/915 | 84/136 | 0·64 (0·47-0·86) |
| Coura-Filho et al., 1994 [[3](#_ENREF_3)] | Descriptive survey in Peri-Peri, Brazil | 480  *(everyone in the study areas was eligible for inclusion, except long-term hospital patients, children below 1 year of age, and the disabled)* | Sewer connection and/or latrine | **Diagnostics:** +½  **Number of samples:** +^1^/_3_  **WASH assessment:** 0  **WASH definitions:** 0  **Confounding assessment:** 0  **Reponse rates:** 0  **Other:** 0  **Total:** +^5^/_6_ | 2x2 table | Barbosa | Sewer connection and/or latrine  Adults and children  South America | 4/15 | 36/88 | 0·65 (0·2-2·1) |
|  |  |  |  |  |  | Peri Peri | Sewer connection and/or latrine  Adults and children  South America | 9/14 | 45/269 | 3·84 (1·57-9·4) |
| Cundill et al., 2011 [[4](#_ENREF_4)] | Baseline from longitudinal study of factors associated with reinfection with *S. mansoni* in Americaninhas, Brazil | 598*  *(people included if they were aged over 5 years, included in the baseline survey, and met other inclusion criteria)* | Latrine | **Diagnostics:** +^1^/_2_  **Number of samples:** +1  **WASH assessment:** 0  **WASH definitions:** 0  **Confounding assessment:** +1  **Reponse rates:** 0  **Other:** 0  **Total:** +2½ | 2x2 table | - | Latrine  Adults and children  South America | 39/286 | 30/243 | 1·10 (0·67-1·83) |
| da Silva et al., 1997 [[5](#_ENREF_5)] | Cross-sectional descriptive survey in Serrano, Cururupu, Brazil | 294 *(systematically sampled from the population of Serrano)* | Cesspool/Septic tank | **Diagnostics:** +½  **Number of samples:** 0  **WASH assessment:** 0  **WASH definitions:** 0  **Confounding assessment:** 0  **Reponse rates:** 0  **Other:** 0  **Total:** +½ | 2x2 table | - | Septic tank/cesspool Adults and children  South America | 69/213 | 2/10 | 1·62 (0·35-7·57) |
| Farooq et al., 1966 [[6](#_ENREF_6)] | Descriptive survey in the Egypt-49 project area, Egypt | 23,572  *(random sample of individuals living in the areas)* | Latrine | **Diagnostics:** +1  **Number of samples:** +^1^/_3_  **WASH assessment:** 0  **WASH definitions:** 0  **Confounding assessment:** +1  **Reponse rates:** +1  **Other:** 0  **Total:** +3^1^/_3_ | 2x2 table | Area 1 - Project area | Latrine  Adults and children  Africa | 1211/6224 | 1475/2876 | 0·38 (0·35-0·41) |
|  |  |  |  |  |  | Area 2 - Rural division | Latrine  Adults and children  Africa | 223/1357 | 681/1889 | 0·46 (0·39-0·54) |
|  |  |  |  |  |  | Area 3 - Urban division | Latrine  Adults and children  Africa | 461/3046 | 34/41 | 0·18 (0·11-0·29) |
|  |  |  |  |  |  | Area 4 - Reclamation division | Latrine  Adults and children  Africa | 177/1346 | 81/201 | 0·33 (0·24-0·44) |
|  |  |  |  |  |  | Area 5 - Control division | Latrine  Adults and children  Africa | 350/475 | 678/746 | 0·81 (0·68-0·96) |
| Firmo et al., 1996 [[7](#_ENREF_7)] | Case-control survey in Gorduras, Minas Gerais, Brazil | 916*  *(households selected randomly and all residents in these households were eligible for inclusion)* | Sewerage | **Diagnostics:** +½  **Number of samples:** +^2^/_3_  **WASH assessment:** 0  **WASH definitions:** +1  **Confounding assessment:** 0  **Reponse rates:** +1  **Other:** 0  **Total:** +2^5^/_6_ | 2x2 table | - | Sewerage  Adults and children  South America | 408/421 | 43/44 | 0·99 (0·64-1·54) |
| Fürst et al., 2013 [[8](#_ENREF_8)] | Descriptive survey as part of the Taabo health demographic surveillance system, in south-central Côte d'Ivoire | 195  *(adults in a stratified random sample of approximately 7% of households in the area of the Taabo health demographic surveillance system)* | Latrine with cement floor or flush toilet | **Diagnostics:** +½  **Number of samples:** +^1^/_3_  **WASH assessment:** 0  **WASH definitions:** +1  **Confounding assessment:** 0  **Reponse rates:** 0  **Other:** 0  **Total:** +1^5^/_6_ | 2x2 table supplied by the authors | - | Latrine or flush toilet  Adults  Africa | 1/66 | 3/125 | 0·63 (0·06-6·19) |
| Guimarães et al., 1985a [[9](#_ENREF_9)] | Descriptive survey in Tuparece, Brazil | 745*  *(all 830 residents registered in the census were eligible for inclusion)* | Latrine | **Diagnostics:** +½  **Number of samples:** +^1^/_3_  **WASH assessment:** 0  **WASH definitions:** 0  **Confounding assessment:** 0  **Reponse rates:** +1  **Other:** 0  Total: +1^5^/_6_ | 2x2 table | - | Latrine  Adults and children  South America | 198/243 | 129/175 | 1·11 (0·82-1·48) |
| Guimarães et al., 1985b [[10](#_ENREF_10)] | Descriptive survey of school children in Ilha, Brazil | 167*  *(all children at the community’s school were eligible for inclusion)* | Latrine | **Diagnostics:** +1  **Number of samples:** +^1^/_3_  **WASH assessment:** 0  **WASH definitions:** 0  **Confounding assessment:** 0  **Reponse rates:** +1  **Other:** 0  **Total:** +2^1^/_3_ | 2x2 table | - | Latrine  Children  South America | 61/95 | 7/4 | 0·37 (0·10-1·31) |
| Kabatereine et al., 2011 [[11](#_ENREF_11)] | Descriptive survey at Lake Victoria, Uganda | 1784*  *(15 children were randomly selected in each village)* | Latrine | **Diagnostics:** +1  **Number of samples:** +^1^/_3_  **WASH assessment:** 0  **WASH definitions:** 0  **Confounding assessment:** 0  **Reponse rates:** +1  **Other:** 0  **Total:** +2^1^/_3_ | Odds ratio from multivariate stepwise logistic regresion model | - | Latrine  Children  Africa | - | - | 0·50 (0·39-0·64) |
| Matthys et al., 2007 [[12](#_ENREF_12)] | Cross-sectional descriptive survey in Man, Côte d'Ivoire | 716  *(households randomly selected and all people in these households were eligible for inclusion)* | Latrine | **Diagnostics:** +1  **Number of samples:** +^1^/_3_  **WASH assessment:** 0  **WASH definitions:** 0  **Confounding assessment:** 0  **Reponse rates:** 0  **Other:** 0  **Total:** +1^1^/_3_ | Odds ratio from bivariate model | - | Latrine  Adults and children  Africa | - | - | 0·51 (0·32-0·81) |
| Mahmud et al., 2013 [[13](#_ENREF_13)] | Survey of schoolchildren in 12 schools in northern Ethiopia | 600  *(schoolchildren randomly selected from school rosters)* | Latrine | **Diagnostics:** +^1^/_2_  **Number of samples:** +^2^/_3_  **WASH assessment:** 0  **WASH definitions:** 0  **Confounding assessment:** 0  **Reponse rates:** +1  **Other:** 0  **Total:** +4^1^/_3_ | 2x2 table | - | Latrine  Children  Africa | 26/238 | 54/282 | 0·57 (0·35-0·94) |
| Noman et al., 2012 [[14](#_ENREF_14)] | Descriptive survey in schoolchildren in Taiz governorate, Yemen | 210  *(not specified)* | Toilet | **Diagnostics:** +½  **Number of samples:** 0  **WASH assessment:** 0  **WASH definitions:** 0  **Confounding assessment:** 0  **Reponse rates:** 0  **Other:** 0  **Total:** +½ | 2x2 table | - | Latrine or flush toilet  Children  Asia | 57/115 | 14/24 | 0·85 (0·41-1·77) |
| Palmeira et al., 2010 [[15](#_ENREF_15)] | Survey of schoolchildren in two municpalities in the state of Alagos, Brazil | 329*  *(all school children aged 7-15 years in the two municipalities studied were eligible for inclusion)* | Pit latrine or sewerage | **Diagnostics:** +½  **Number of samples:** +^2^/_3_  **WASH assessment:** 0  **WASH definitions:** 0  **Confounding assessment:** +1  **Reponse rates:** 0  **Other:** 0  **Total:** +2^1^/_6_ | 2x2 table | - | Latrine or flush toilet  Children  South America | 47/171 | 13/38 | 0·80 (0·40-1·63) |
| Rodrigues et al., 1995 [[16](#_ENREF_16)] | Descriptive survey in Itinga, Brazil | 324*  *(all inhabitants of the village of Ponte Do Pasmado were eligible for inclusion)* | Cesspool/Septic tank | **Diagnostics:** +1  **Number of samples:** +^1^/_3_  **WASH assessment:** 0  **WASH definitions:** 0  **Confounding assessment:** 0  **Reponse rates:** +1  **Other:** 0  **Total:** +2^1^/_3_ | 2x2 table | - | Septic tank/cesspool  Adults and children  South America | 73/85 | 90/76 | 0·73 (0·47-1·12) |
| Sady et al., 2013 [[17](#_ENREF_17)] | Decsriptive survey in 10 districts in western Yemen | 400  *(households randomly selected and all children up to 15 years of age were eligible for inclusion in the study)* | Presence of toilet in house | **Diagnostics:** +1  **Number of samples:** 0  **WASH assessment:** 0  **WASH definitions:** 0  **Confounding assessment:** 0  **Reponse rates:** 0  **Other:** 0  **Total:** +1 | 2x2 table supplied by the authors | - | Latrine or flush toilet  Children  Asia | 7/213 | 30/150 | 0·16 (0·07-0·38) |
| Ximenes et al., 2003 [[18](#_ENREF_18)] | Cross-sectional descriptive survey in São Lourenço da Mata, Brazil | 1674 families*  *(households randomly selected and all those aged 10-25 years in those households were eligible for inclusion)* | Sewerage | **Diagnostics:** +½  **Number of samples:** 0  **WASH assessment:** 0  **WASH definitions:** 0  **Confounding assessment:** +1  **Reponse rates:** 0  **Other:** 0  **Total:** +1^1^/_2_ | Odds ratios from biavariate models | Pit or septic tank | Sewerage  Adults and children  Latrine or flush toilet | - | - | 0·60 (0·44-0·84) |
|  |  |  |  |  |  | Sewerage | Sewerage  Adults and children  South America | - | - | 0·20 (0·14-0·29) |

* WASH and schistosomiasis data not available for complete study population. Population reported is the number with WASH and schistosomiasis data available

^†^ Quality of studies was assessed by assigning scores for diagnostic approach (+1 if sedimentation was used for intestinal schistosomiasis or multiple diagnostic approaches used, +1/2 for Kato-Katz or urine filtration, 0 otherwise); number of samples analysed (+1 if multiple stool/urine samples taken, +2/3 if slides checked by another technician, +1/3 if multiple slides read from the same sample, 0 otherwise); WASH assessment (+1 if household visit and inspection, or at least some spot checks, 0 if questionnaire outside of the home or WASH assessment method not defined); WASH definitions (+1 if they allow for comparison with JMP indicators,[[19](#_ENREF_19)] 0 otherwise); confounding assessment (+1 for data being split according to non-WASH variables found to be predictive of infection, 0 otherwise); response rates (+1 for above 80%, 0 for below 80% or not defined); and other (+1 for additional strengths and -1 for additional weaknesses).

## References

1. Barbosa FA (1966) [Schistosomiasis morbidity]. R Br Malar: Suppl:3-159. (in Portuguese)

2. Barreto ML (1991) Geographical and socioeconomic factors relating to the distribution of *Schistosoma mansoni* infection in an urban area of north-east Brazil. Bull World Health Organ 69: 93-102.

3. Coura-Filho P, Rocha RS, Farah MW, da Silva GC, Katz N (1994) Identification of factors and groups at risk of infection with *Schistosoma mansoni*: a strategy for the implementation of control measures? Rev Inst Med Trop São Paulo 36: 245-253.

4. Cundill B, Alexander N, Bethony JM, Diemert D, Pullan RL, et al. (2011) Rates and intensity of re-infection with human helminths after treatment and the influence of individual, household, and environmental factors in a Brazilian community. Parasitology 138: 1406-1416.

5. da Silva AA, Cutrim RN, de Britto e Alves MT, Coimbra LC, Tonial SR, et al. (1997) Water-contact patterns and risk factors for *Schistosoma mansoni* infection in a rural village of northeast Brazil. Rev Inst Med Trop São Paulo 39: 91-96.

6. Farooq M, Nielsen J, Samaan SA, Mallah MB, Allam AA (1966) The epidemiology of *Schistosoma haematobium* and *S. mansoni* infections in the Egypt-49 project area. 2. Prevalence of bilharziasis in relation to personal attributes and habits. Bull World Health Organ 35: 293-318.

7. Firmo JOA, Costa MF, Guerra HL, Rocha RS (1996) Urban schistosomiasis: morbidity, sociodemographic characteristics and water contact patterns predictive of infection. Int J Epidemiol 25: 1292-1300.

8. Fürst T, Ouattara M, Silué KD, N'Goran DN, Adiossan LG, et al. (2013) Scope and limits of an anamnestic questionnaire in a control-induced low-endemicity helminthiasis setting in south-central Côte d'Ivoire. PLoS One 8: e64380.

9. Guimarães DC, De Barros HL, Katz N (1985) A clinical epidemiologic study in a schistosomiasis mansoni endemic area. Rev Inst Med Trop São Paulo 27: 123-131.

10. Guimarães MD, Costa MF, de Lima LB, Moreira MA (1985) [Clinico-epidemiological study of schistosomiasis mansoni in school children of Ilha, municipality of Arcos, MG (Brazil) 1983]. Rev Saude Publica 19: 8-17. (in Portuguese)

11. Kabatereine NB, Standley CJ, Sousa-Figueiredo JC, Fleming FM, Stothard JR, et al. (2011) Integrated prevalence mapping of schistosomiasis, soil-transmitted helminthiasis and malaria in lakeside and island communities in Lake Victoria, Uganda. Parasit Vectors 4: 232.

12. Matthys B, Tschannen AB, Tian-Bi NT, Comoe H, Diabate S, et al. (2007) Risk factors for *Schistosoma mansoni* and hookworm in urban farming communities in western Côte d’Ivoire. Trop Med Int Health 12: 709-723.

13. Mahmud MA, Spigt M, Bezabih AM, Lopez Pavon I, Dinant G-J, et al. (2013) Risk factors for intestinal parasitosis, anaemia, and malnutrition among school children in Ethiopia. Pathog Glob Health 107: 58-65.

14. Noman MA, Alshargby SA, Kadi HO, Mansoor T, Rahman RA, et al. (2012) Spread of internal parasites among the student of Al-Shaheed Al-Noman school, Widi Jadeed villages, Taiz province-Republic of Yemen. C J BioMed 6: 18-24.

15. Palmeira DCC, de Carvalho AG, Rodrigues K, Couto JLA (2010) [Prevalence of *Schistosoma mansoni* infection in two municipalities of the State of Alagoas, Brazil]. Rev Soc Bras Med Trop 43: 313-317. (in Portuguese)

16. Rodrigues RN, Murta C, Teixeira Junior MA, Cury GC, Rocha MO (1995) Clinical-epidemiologic study of schistosomiasis mansoni in Ponte do Pasmado, a village in the municipality of Itinga, state of Minas Gerais, Brazil, 1992. Rev Inst Med Trop São Paulo 37: 81-85.

17. Sady H, Al-Mekhlafi HM, Mahdy MAK, Lim YAL, Mahmud R, et al. (2013) Prevalence and associated factors of schistosomiasis among children in Yemen: implications for an effective control programme. PLoS Negl Trop Dis 7: e2377.

18. Ximenes R, Southgate B, Smith PG, Guimaraes Neto L (2003) Socioeconomic determinants of schistosomiasis in an urban area in the Northeast of Brazil. Rev Panam Salud Publica 14: 409-421.

19. WHO, UNICEF (2013) Progress on sanitation and drinking-water - 2013 update. Geneva: World Health Organization.
