# Supplementary material for: The Relationship between Water, Sanitation and Schistosomiasis: A Systematic Review and Meta-analysis
Source: PLoS Negl Trop Dis. 2014 Dec 4;8(12):e3296. doi: 10.1371/journal.pntd.0003296 (PMC4256273; doi:10.1371/journal.pntd.0003296)
Supplement: Table S4 — Included study characteristics for the adequate sanitation and S. haematobium meta-analysis. (DOCX) [file pntd.0003296.s007.docx]

### Included Study Characteristics for the Adequate Sanitation and *S. haematobium* Meta-analysis

| **Reference** | **Study design, setting** | **Study population *(selection)*** | **‘Adequate’ sanitation definition** | **Study quality assessment^†^** | **Data obtained** | **Dataset** | **Sub-analyses categories**  *(type of sanitation,*  *continent)* | **Odds of infection in those with ‘improved sanitation’**  *(number infected with improved sanitation /number uninfected with improved sanitation)* | **Odds of infection in those without ‘improved sanitation’**  *(number infected with unimproved sanitation /number uninfected without improved sanitation)* | **OR (CI)** |
| --- | --- | --- | --- | --- | --- | --- | --- | --- | --- | --- |
| Abou-Zeid et al., 2012 [[1](#_ENREF_1)] | Cross-sectional descriptive survey in adults in South Kordofan state, Sudan | 1826  *(households randomly selected and all people above 18 years of age and in those households were eligible for inclusion)* | Latrine | **Diagnostics:** +½  **Number of samples:** +^1^/_3_  **WASH assessment:** 0  **WASH definitions:** 0  **Confounding assessment:** 0  **Reponse rates:** +1  **Other:** 0  **Total:** +1^5^/_6_ | 2x2 table | - | Latrine  Adults  Africa | 65/948 | 61/752 | 0·85 (0·59-1·21) |
| Al-Waleedi et al., 2013 [[2](#_ENREF_2)] | Descriptive survey in schoolchildren in Abyan governorate, Yemen | 696  *(all schoolchildren in the village)* | Septic tank or cesspool | **Diagnostics:** +½  **Number of samples:** 0  **WASH assessment:** 0  **WASH definitions:** 0  **Confounding assessment:** 0  **Reponse rates:** +1  **Other:** 0  **Total:** +1^1^/_2_ | 2x2 table | - | Septic tank or cesspool  Children  Asia | 99/488 | 27/82 | 0·62 (0·38-1·00) |
| Awoke et al., 2013 [[3](#_ENREF_3)] | Descriptive survey in purposively sampled schools in Amibera district, Ethiopia | 832  *(randomly selected from the purposively sampled schools)* | Toilet utilization | **Diagnostics:** +1  **Number of samples:** +^2^/_3_  **WASH assessment:** 0  **WASH definitions:** 0  **Confounding assessment:** 0  **Reponse rates:** +1  **Other:** 0  **Total:** +2^2^/_3_ | 2x2 table | - | Toilet  Children  Africa | 28/486 | 33/285 | 0·50 (0·29-0·84) |
| Farooq et al., 1966 [[4](#_ENREF_4)] | Descriptive survey in the Egypt-49 project area, Egypt | 23572  *(random sample of individuals living in the areas)* | Latrine | **Diagnostics:** +1  **Number of samples:** +^1^/_3_  **WASH assessment:** 0  **WASH definitions:** 0  **Confounding assessment:** +1  **Reponse rates:** +1  **Other:** 0  **Total:** +3^1^/_3_ | 2x2 table | Area 1 – Project area | Latrine  Adults and children  Africa | 1566/5869 | 1479/2872 | 0·52 (0·48-0·56) |
|  |  |  |  |  |  | Area 2 - Rural division | Latrine  Adults and children  Africa | 386/1194 | 766/1804 | 0·76 (0·66-0·88) |
|  |  |  |  |  |  | Area 3 - Urban division | Latrine  Adults and children  Africa | 431/3076 | 25/50 | 0·28 (0·17-0·46) |
|  |  |  |  |  |  | Area 4 - Reclamation division | Latrine  Adults and children  Africa | 446/1077 | 91/191 | 0·87 (0·66-1·14) |
|  |  |  |  |  |  | Area 5 - Control division | Latrine  Adults and children  Africa | 300/525 | 597/827 | 0·79 (0·66-0·94) |
| Noman et al., 2012 [[5](#_ENREF_5)] | Descriptive survey in schoolchildren in Taiz governorate, Yemen | 210  *(not specified)* | Toilets | **Diagnostics:** +½  **Number of samples:** 0  **WASH assessment:** 0  **WASH definitions:** 0  **Confounding assessment:** 0  **Reponse rates:** 0  **Other:** 0  **Total:** +½ | 2x2 table | - | Latrine or flush toilet  Children  Asia | 15/159 | 4/32 | 0·75 (0·24-2·42) |
| Knopp et al., 2013a [[6](#_ENREF_6)] | Descriptive study in Bandmaji and Dole, Unguja, Zanzibar | 382*  *(everyone aged 5 years and above eligible for inclusion)* | Latrine | **Diagnostics:** +½  **Number of samples:** +^2^/_3_  **WASH assessment:** 0  **WASH definitions:** 0  **Confounding assessment:** 0  **Reponse rates:** 0  **Other:** 0  **Total:** +^5^/_6_ | 2x2 table supplied by author | - | Latrine  Adults and children  Africa | 27/270 | 4/81 | 2·03 (0·69-5·96) |
| Sady et al., 2013 [[7](#_ENREF_7)] | Decsriptive survey in 10 districts in western Yemen | 400  *(households randomly selected and all children up to 15 years of age were eligible for inclusion in the study)* | Presence of toilet in house | **Diagnostics:** +1  **Number of samples:** 0  **WASH assessment:** 0  **WASH definitions:** 0  **Confounding assessment:** 0  **Reponse rates:** 0  **Other:** 0  **Total:** +1 | 2x2 table supplied by the authors | - | Latrine or flush toilet  Children  Asia | 49/171 | 46/134 | 0·83 (0·53-1·32) |
| Stephenson et al., 1986 [[8](#_ENREF_8)] | Case-control survey in Kwale district, Kenya | 105  *(51 positive and 54 negative children, matched for age and sex)* | Latrine | **Diagnostics:** +½  **Number of samples:** +1  **WASH assessment:** 0  **WASH definitions:** 0  **Confounding assessment:** +1  **Reponse rates:** +1  **Other:** 0  **Total:** +1^1^/_2_ | 2x2 table | - | Latrine  Children  Africa | 15/15 | 36/39 | 1·08 (0·46-2·53) |

* WASH and schistosomiasis data not available for complete study population. Population reported is the number with WASH and schistosomiasis data available

^†^ Quality of studies was assessed by assigning scores for diagnostic approach (+1 if sedimentation was used for intestinal schistosomiasis or multiple diagnostic approaches used, +1/2 for Kato-Katz or urine filtration, 0 otherwise); number of samples analysed (+1 if multiple stool/urine samples taken, +2/3 if slides checked by another technician, +1/3 if multiple slides read from the same sample, 0 otherwise); WASH assessment (+1 if household visit and inspection, or at least some spot checks, 0 if questionnaire outside of the home or WASH assessment method not defined); WASH definitions (+1 if they allow for comparison with JMP indicators,[[9](#_ENREF_9)] 0 otherwise); confounding assessment (+1 for data being split according to non-WASH variables found to be predictive of infection, 0 otherwise); response rates (+1 for above 80%, 0 for below 80% or not defined); and other (+1 for additional strengths and -1 for additional weaknesses).

## References

1. Abou-Zeid AHA, Abkar TA, Mohamed RO (2012) Schistosomiasis and soil-transmitted helminths among an adult population in a war affected area, Southern Kordofan state, Sudan. Parasit Vectors 5: 133.

2. Al-Waleedi AA, El-Nimr NA, Hasab AA, Bassiouny HK, Al-Shibani LA (2013) Urinary schistosomiasis among schoolchildren in Yemen: prevalence, risk factors, and the effect of a chemotherapeutic intervention. J Egypt Public Health Assoc 88: 130-136.

3. Awoke W, Bedimo M, Tarekegn M (2013) Prevalence of schistosomiasis and associated factors among students attending at elementary schools in Amibera District, Ethiopia. Open J Prev Med 3: 199-204.

4. Farooq M, Nielsen J, Samaan SA, Mallah MB, Allam AA (1966) The epidemiology of *Schistosoma haematobium* and *S. mansoni* infections in the Egypt-49 project area. 2. Prevalence of bilharziasis in relation to personal attributes and habits. Bull World Health Organ 35: 293-318.

5. Noman MA, Alshargby SA, Kadi HO, Mansoor T, Rahman RA, et al. (2012) Spread of internal parasites among the student of Al-Shaheed Al-Noman school, Widi Jadeed villages, Taiz province-Republic of Yemen. C J BioMed 6: 18-24.

6. Knopp S, Stothard JR, Rollinson D, Mohammed KA, Khamis IS, et al. (2013) From morbidity control to transmission control: time to change tactics against helminths on Unguja Island, Zanzibar. Acta Trop 128: 412-422.

7. Sady H, Al-Mekhlafi HM, Mahdy MAK, Lim YAL, Mahmud R, et al. (2013) Prevalence and associated factors of schistosomiasis among children in Yemen: implications for an effective control programme. PLoS Negl Trop Dis 7: e2377.

8. Stephenson LS, Elliot TC, Kinoti SN (1986) Water, sanitation and knowledge about urinary schistosomiasis in a Kenyan coastal community: a study combining ethnographic and survey techniques. In: Stephenson LS, editor. Schistosomiasis and Malnutrition. Ithaca: Cornell University, Division of Nutritional Sciences. pp. 69-192.

9. WHO, UNICEF (2013) Progress on sanitation and drinking-water - 2013 update. Geneva: World Health Organization.
