# Supplementary material for: The Relationship between Water, Sanitation and Schistosomiasis: A Systematic Review and Meta-analysis
Source: PLoS Negl Trop Dis. 2014 Dec 4;8(12):e3296. doi: 10.1371/journal.pntd.0003296 (PMC4256273; doi:10.1371/journal.pntd.0003296)
Supplement: Text S1 — Study protocol. (DOCX) [file pntd.0003296.s008.docx]

# Study protocol for systematic review and meta-analysis of the relationship between water, sanitation and schistosomiasis

## Objective

To examine the effect of water, sanitation and hygiene on human schistosome infection.

## Authors

This study will be conducted primarily by Jack E. T. Grimes (“JETG”) (MSc) with support from David Croll (“DC”) (MSc) and leadership from Michael R. Templeton (PhD), Wendy E. Harrison, (PhD), Jürg Utzinger (“JU”) (PhD), and Matthew Freeman (PhD).

## Reporting

We will adhere to the Preferred Reporting Items for Systematic Reviews and Meta-Analyses (PRISMA) statement ([Moher et al., 2009](#_ENREF_4)), as well as the Meta-analysis of Observational Studies in Epidemiology (MOOSE) guidelines ([Stroup et al., 2000](#_ENREF_6)).

## Search methods

We will search PubMed, Web of Science, Embase and the Cochrane Library for this review. We will also scan the bibliographies of several recent systematic reviews ([Ziegelbauer et al., 2012](#_ENREF_9), [Stocks et al., 2014](#_ENREF_5), [Strunz et al., 2014](#_ENREF_7)). We will consider papers published at any time before 31 December 2013, and in any language. If a study under consideration cites another paper that appears relevant, the second study will also be reviewed and considered for inclusion. Our searches will include each of the following WASH-related terms in conjunction with each of the following schistosome-related terms:

**WASH terms:**

water, borehole, standpipe, rainwater, sanitation, sanitary engineering, latrine, toilet, pit, open defecation, open urination, shower, laundry, hygiene, detergent, soap, risk factor

**Schistosome terms:**

schistosomiasis, schistosome, schistosoma, bilharzia, bilharziasis, snail fever

The search terms will be combined as follows: (schistosomiasis OR schistosome OR schistosoma OR bilharzia OR bilharziasis OR snail fever) AND (water OR borehole OR standpipe OR rainwater OR sanitation OR sanitary engineering OR latrine OR toilet OR pit OR open defecation OR open urination OR shower OR laundry OR hygiene OR detergent OR soap OR risk factor).

## Study inclusion criteria

Included studies must have assessed WASH, assessed schistosome infection, and compared the two. We expect observational studies to form the largest group of comparable studies, and we therefore plan to carry out meta-analyses of such studies. However, we will consider any form of study to see if meta-analyses of evidence reported in different formats are possible.

Where a study has combined schistosome infection with other parasites (including other schistosome species) as an outcome measure, the data on individual schistosome species will be requested. If this is not available, then the study will be excluded. Similarly, if WASH facilities have been combined, the data will be requested for individual WASH facilities. If this is not available, the study will be excluded.

Studies will not be excluded according to participants, who could be of any age or from different occupational groups. Schistosomiasis will be defined as infection with any human schistosome, assessed through the presence of eggs in the urine or stool. Water will be defined as the use or availability of a community or household source considered ‘safe’ by the study authors, while adequate sanitation will be defined as the presence of a latrine, septic tank, cesspit or sewerage connection in the household.

JETG and DC will independently review the studies returned by the searches. First the titles will be reviewed to exclude papers obviously not about WASH, not about human schistosomiasis or about computer models (rather than data collected from the field). Next, abstracts will be reviewed, and papers will be excluded according to the same criteria, but also when papers obviously have a large confounder such as chemotherapy which limits what may be said about the impact of WASH in those studies. Finally, the full texts will be reviewed and papers excluded according to the previous criteria, or included in the meta-analyses. At each stage, papers will be classified by JETG and DC independently. Disagreements will be discussed until consensus is reached. Where disagreements cannot be resolved, a third reviewer (JU) will decide whether or not the studies should be included.

Odds ratios will be used as the effect measure in all meta-analyses. Studies reporting odds ratios according to WASH status directly, or 2x2 data from which they can be calculated, will be eligible for inclusion. Additionally, studies reporting the sensitivity, specificity, and positive predictive value (PPV) of WASH variables for schistosome infection will be eligible for inclusion, since the 2x2 tables may be derived from such equations. If studies indicate that useful data has been collected but not reported, the authors will be contacted, and the useful data requested. Studies with an odds ratio of less than 1 indicate lower levels of schistosome infection among those with access to the form of WASH under consideration. Where a study reports multiple datasets or effect sizes from different settings, these will all be eligible for inclusion. If a 2x2 table contains 0 in one of the elements, it will be excluded due to the problematic behaviour this causes in the calculation of the odds ratio. Where a study reports effect sizes for different kinds of water, sanitation or hygiene, all effect sizes will be included in the meta-analyses; the double-counting of certain participants is thought to be preferable to the bias induced by choosing which effect size to include.

## Data collection

Data in the form of 2x2 tables or odds ratios with confidence intervals (CIs) will be extracted independently by JETG and DC, with errors being discussed and corrected. The data will be extracted as follows: from 2x2 tables (of *a* = number infected with WASH, *b* = number uninfected with WASH, *c* = number infected without WASH, *d* = number uninfected without WASH) the standard error (SE) will be calculated as $\sqrt{\frac{1}{a}+\frac{1}{b}+\frac{1}{c}+\frac{1}{d}}$, where *a*, *b*, *c*, and *d* represent the four cells of the 2x2 table. From odds ratios and CIs, the SE will be calculated as $\frac{\ln\left( upper confidence limit \right)-\ln\left( OR \right)}{1\cdot96}$. The odds ratios will be calculated from 2x2 tables as $\frac{a/b}{c/d}$, where $\frac{a}{b}$ is the odds of infection among those with access to improved WASH, and $\frac{c}{d}$ is the odds of infection among those without access to improved WASH. Some papers may report odds ratios for infection, with improved WASH as the baseline. In these cases, the inverses of the reported odds ratios and CIs will be taken to ensure consistency. Thus odds ratios below 1 will denote reduced odds of infection in those with improved WASH.

Additional information on the included studies (study design, setting, year, schistosome species, type of WASH, study population and selection, sample size, assessment of WASH, and diagnostics used will be extracted by JETG and cross-checked by DC.

## Quality assessment

Included studies’ quality will be assessed though an approach adapted from the GRADE methodology ([Atkins et al., 2004](#_ENREF_1)), and previous similar reviews ([Ziegelbauer et al., 2012](#_ENREF_9), [Stocks et al., 2014](#_ENREF_5), [Strunz et al., 2014](#_ENREF_7)). Studies will be assessed according to diagnostic approach (with sedimentation for intestinal schistosomiasis being rewarded due to its higher sensitivity compared with a single Kato-Katz reading), collection of multiple samples, WASH assessment, WASH definitions, confounding assessment, response rates, and other strengths and limitations (Table 1).

Table 1: Quality assessment of included studies

| **Diagnostic approach** | |
| --- | --- |
| Sedimentation for intestinal schistosomiasis or multiple diagnostic approaches used | +1 |
| Kato-Katz or urine filtration | +1/2 |
| Other diagnostics | 0 |
| **Number of samples analysed** | |
| Multiple stool samples taken | +1 |
| Slides checked by another technician | +2/3 |
| Multiple slides prepared from the same sample | +1/3 |
| Other number of samples analysed | 0 |
| **WASH assessment** | |
| Household visit and inspection or at least some spot checks | +1 |
| Questionnaire outside of home or WASH assessment not defined | 0 |
| **WASH definitions** | |
| Allow for comparison with WHO, UNICEF JMP indicators ([WHO and UNICEF, 2013](#_ENREF_8)) | +1 |
| Don’t allow for comparison | 0 |
| **Confounding assessment** | |
| Data split according to non-WASH variables found to be predictive of infection | +1 |
| Data not split as above | 0 |
| **Response rates** | |
| Above 80% for both WASH and schistosomiasis data | +1 |
| Below 80% for both WASH and schistosomiasis, or undefined | 0 |
| **Additional strengths or weaknesses** | |
| Additional strengths | +1 |
| Additional weaknesses | -1 |

JETG and DC will independently assess included studies quality, and any disagreements will be discussed with a third reviewer (JU) until consensus is reached.

## Meta-analysis

Separate meta-analyses will be conducted for water and schistosomiasis, sanitation and intestinal schistosomiasis, and sanitation and urogenital schistosomiasis. Random effects models ([DerSimonian and Laird, 1986](#_ENREF_2)) in StatsDirect 2.8.0 (StatsDirect Ltd, Altrincham, UK) will be used throughout, weighting datasets’ effect sizes by their inverse variances. Egger’s test ([Egger et al., 1997](#_ENREF_3)) and visual inspection of funnel plots will be used to assess publication bias of the main meta-analyses.

Higgins’ *I^2^* will be used to assess heterogeneity of the meta-analyses, and where this is high (>75%), and it is feasible, sub-analyses will investigate different subsets of studies. These will investigate whether splitting the analyses according to the kind of schistosome (intestinal or urogenital), the location of the water source, the type of sanitation, or the continent reduces this heterogeneity.

## References

Atkins, D., Best, D., Briss, P. A., Eccles, M., Falck-Ytter, Y., Flottorp, S., Guyatt, G. H., Harbour, R. T., Haugh, M. C., Henry, D., Hill, S., Jaeschke, R., Leng, G., Liberati, A., Magrini, N., Mason, J., Middleton, P., Mrukowicz, J., O'Connell, D., Oxman, A. D., Phillips, B., Schunemann, H. J., Edejer, T. T., Varonen, H., Vist, G. E., Williams, J. W., Jr. & Zaza, S. 2004. Grading quality of evidence and strength of recommendations. *Bmj,* 328**,** 1490.

DerSimonian, R. & Laird, N. 1986. Meta-analysis in clinical trials. *Control Clin Trials,* 7**,** 177-188.

Egger, M., Smith, G. D., Schneider, M. & Minder, C. 1997. Bias in meta-analysis detected by a simple, graphical test. *BMJ,* 315**,** 629-634.

Moher, D., Liberati, A., Tetzlaff, J. & Altman, D. G. 2009. Preferred reporting items for systematic reviews and meta-analyses: the PRISMA statement. *PLoS Med,* 6**,** e1000097.

Stocks, M. E., Ogden, S., Haddad, D., Addiss, D. G., McGuire, C. & Freeman, M. C. 2014. Effect of water, sanitation, and hygiene on the prevention of trachoma: a systematic review and meta-analysis. *PLoS Med,* 11**,** e1001605.

Stroup, D. F., Berlin, J. A., Morton, S. C., Olkin, I., Williamson, G. D., Rennie, D., Moher, D., Becker, B. J., Sipe, T. A. & Thacker, S. B. 2000. Meta-analysis of observational studies in epidemiology: a proposal for reporting. Meta-analysis Of Observational Studies in Epidemiology (MOOSE) group. *JAMA,* 283**,** 2008-12.

Strunz, E. C., Addiss, D. G., Stocks, M., Ogden, S., Utzinger, J. & Freeman, M. C. 2014. Water, sanitation, hygiene, and soil-transmitted helminth infection: a systematic review and meta-analysis. *PLoS Med,* 11**,** e1001620.

WHO & UNICEF 2013. Progress on sanitation and drinking-water - 2013 update. *Geneva: World Health Organization*.

Ziegelbauer, K., Speich, B., Mäusezahl, D., Bos, R., Keiser, J. & Utzinger, J. 2012. Effect of sanitation on soil-transmitted helminth infection: systematic review and meta-analysis. *PLoS Med,* 9**,** e1001162.
