# Supplementary material for: The Relationship between Water, Sanitation and Schistosomiasis: A Systematic Review and Meta-analysis
Source: PLoS Negl Trop Dis. 2014 Dec 4;8(12):e3296. doi: 10.1371/journal.pntd.0003296 (PMC4256273; doi:10.1371/journal.pntd.0003296)
Supplement: Text S2 — MOOSE checklist. (DOC) [file pntd.0003296.s009.doc]

# MOOSE Checklist

From: [Donna F. Stroup](http://jama.ama-assn.org/search?author1=Donna+F.+Stroup&sortspec=date&submit=Submit), PhD, MSc; [Jesse A. Berlin](http://jama.ama-assn.org/search?author1=Jesse+A.+Berlin&sortspec=date&submit=Submit), ScD; [Sally C. Morton](http://jama.ama-assn.org/search?author1=Sally+C.+Morton&sortspec=date&submit=Submit), PhD; [Ingram Olkin](http://jama.ama-assn.org/search?author1=Ingram+Olkin&sortspec=date&submit=Submit), PhD; [G. David Williamson](http://jama.ama-assn.org/search?author1=G.+David+Williamson&sortspec=date&submit=Submit), PhD; [Drummond Rennie](http://jama.ama-assn.org/search?author1=Drummond+Rennie&sortspec=date&submit=Submit), MD; [David Moher](http://jama.ama-assn.org/search?author1=David+Moher&sortspec=date&submit=Submit), MSc; [Betsy J. Becker](http://jama.ama-assn.org/search?author1=Betsy+J.+Becker&sortspec=date&submit=Submit), PhD; [Theresa Ann Sipe](http://jama.ama-assn.org/search?author1=Theresa+Ann+Sipe&sortspec=date&submit=Submit), PhD; [Stephen B. Thacker](http://jama.ama-assn.org/search?author1=Stephen+B.+Thacker&sortspec=date&submit=Submit), MD, MSc; for the Meta-analysis Of Observational Studies in Epidemiology (MOOSE) Group. **Meta-analysis of Observational Studies in Epidemiology. A Proposal for Reporting** JAMA. 2000;283(15):2008-2012. doi: 10.1001/jama.283.15.2008

|  | **Reported** |
| --- | --- |
| **Reporting of background should include** | |
| Problem definition | Introduction, paragraph 5 |
| Hypothesis statement | Introduction, paragraph 3 |
| Description of study outcomes | Methods, paragraph 5 |
| Type of exposure or intervention used | Methods, paragraph 2 |
| Type of study designs used | Methods, paragraph 5 |
| Study population | Methods, paragraph 5 |
| **Reporting of search strategy should include** | |
| Qualifications of searchers (eg librarians and investigators) | Methods, paragraphs 2-5 |
| Search strategy, including time period used in the synthesis and key words | Methods, paragraphs 2-5 |
| Effort to include all available studies, including contact with authors | Methods, paragraphs 2-5 |
| Databases and registries searched | Methods, paragraphs 1-2 |
| Search software used, name and version, including special features used (eg explosion) | Methods, paragraph 2 |
| Use of hand searching (eg reference lists of obtained articles) | Methods, paragraph 4 |
| List of citations located and those excluded, including justification | Text S4 |
| Method of addressing articles published in languages other than English | Methods, paragraph 7 |
| Method of handling abstracts and unpublished studies | Methods, paragraph 6 |
| Description of any contact with authors | Methods, paragraph 4 |
| **Reporting of methods should include** | |
| Description of relevance or appropriateness of studies assembled for assessing the hypothesis to be tested | Methods, paragraphs 1,5 |
| Rationale for the selection and coding of data (eg sound clinical principles or convenience) | Methods, paragraph 5 |
| Documentation of how data were classified and coded (eg multiple raters, blinding and interrater reliability) | Methods, paragraph 6 |
| Assessment of confounding (eg comparability of cases and controls in studies where appropriate) | Methods, paragraph 9 |
| Assessment of study quality, including blinding of quality assessors, stratification or regression on possible predictors of study results | Methods, paragraph 9 |
| Assessment of heterogeneity | Methods, paragraph 12 |
| Description of statistical methods (eg complete description of fixed or random effects models, justification of whether the chosen models account for predictors of study results, dose-response models, or cumulative meta-analysis) in sufficient detail to be replicated | Methods, paragraph 11 |
| Provision of appropriate tables and graphics | Tables, figures |
| **Reporting of results should include** | |
| Graphic summarizing individual study estimates and overall estimate | Figures 2-4 |
| Table giving descriptive information for each study included | Tables 1-3 |
| Results of sensitivity testing (eg subgroup analysis) | Results, paragraphs 9-13, 15-19, 21-24 |
| Indication of statistical uncertainty of findings | Tables 1-3, Figures 2-4 |
| **Reporting of discussion should include** | |
| Quantitative assessment of bias (e.g., publication bias) | Results, paragraphs 8, 15, 21 |
| Justification for exclusion (eg exclusion of non-English language citations) | Text S4 |
| Assessment of quality of included studies | Discussion, paragraph 6-10, Tables 1-3 |
| **Reporting of conclusions should include** | |
| Consideration of alternative explanations for observed results | Discussion, paragraph 6-7 |
| Generalization of the conclusions (eg appropriate for the data presented and within the domain of the literature review) | Discussion, paragraphs 1,4 |
| Guidelines for future research | Discussion, paragraph 8, Conclusions |
| Disclosure of funding source | Funding, abstract |
