# Supplementary material for: The Relationship between Water, Sanitation and Schistosomiasis: A Systematic Review and Meta-analysis
Source: PLoS Negl Trop Dis. 2014 Dec 4;8(12):e3296. doi: 10.1371/journal.pntd.0003296 (PMC4256273; doi:10.1371/journal.pntd.0003296)
Supplement: Alternative Language Abstract S1 — Helfen sauberes Trinkwasser und sanitäre Einrichtungen gegen Bilharziose? Systematische Meta-Analyse und Literatur-Review - Translation of abstract into German by David Croll. (DOCX) [file pntd.0003296.s011.docx]

**Helfen sauberes Trinkwasser und sanitäre Einrichtungen gegen Bilharziose? Systematische Meta-Analyse und Literatur-Review.**

**Zusammenfassung**

***Hintergrund:*** Zugang zu gesundheitlich unbedenklichem Trink- und Brauchwasser sowie angemessenen sanitären Einrichtungen werden seit längerem als wichtige Maßnahmen zur Bekämpfung der Bilharziose angesehen, insbesondere weil der Krankheitserreger für die erfolgreiche Fortpflanzung auf menschlichen Kontakt mit Wasser und auf mangelnde Hygiene angewiesen ist. Allerdings wurde der Nutzen sauberen Trinkwassers und sanitärer Einrichtungen bislang noch nie mittels systematischer Literaturrecherche und einer Meta-Analyse aufgearbeitet.

***Methodologie:*** Wir haben verschiedene elektronische Datenbanken (PubMed, Web of Science, Embase und Cochrane Library) systematisch nach Studien, die zu diesem Thema durchgeführt wurden, durchsucht. Die Einschlusskriterien umfassten: Angabe von Infektionsraten in Populationen mit und ohne Zugang zu unbedenklichem Wasser beziehungsweise angemessene sanitäre Einrichtungen, und das Erscheinen in einer der Datenbanken vor dem Jahr 2014. Keine Studie wurde aufgrund des Publikationsdatums oder der Sprache ausgeschlossen. Die Titel und Zusammenfassungen aller Suchresultate wurden unabhängig von zwei Mitarbeitern durchgesehen. Studien, die nicht mit Sicherheit ausgeschlossen werden konnten, wurden im Volltext gelesen. Brauchbare Publikationen wurden schließlich für die Meta-Analyse verwendet. Mittels „Funnel Plots“ und dem Egger-Test wurde eine Verzerrung durch Publikations-Bias bestimmt. Die Heterogenität der Daten wurde mit Higgins-I² gemessen.

***Wichtigste Ergebnisse:*** Verbesserte Wasserqualität ging mit deutlich tieferen Ansteckungsrisiken für Schistosomen einher (odds ratio = 0.53, 95%-Vertrauensintervall 0.47 – 0.61). Angemessene sanitäre Eintrichtungen reduzierten die Infektionsraten mit *Schistosoma mansoni* (OR 0.59, 95% CI: 0.47 – 0.73) und *Schistosoma haematobium* (OR 0.69, 95% CI 0.57 – 0.84). Die ausgewerteten Studien waren zumeist Querschnittsstudien und die Qualität war oftmals gering.

***Schlussfolgerungen/Bedeutung:*** Unsere systematische Literaturrecherche und Meta-Analyse zeigt, dass verbesserter Zugang zu gesundheitlich unbedenklichem Trink- und Brauchwasser und angemessene sanitäre Einrichtungen wichtige Massnahmen zur Bekämpfung der Bilharziose darstellen. Allerdings waren die meisten ausgewerteten Arbeiten beobachtend und beinhalteten keine Interventionen. Dazu war die methodische Qualität meist gering. Daraus ergibt sich ein dringender Bedarf an methodisch verbesserten, randomisierten Studien, und ebenso Studien, welche die untersuchten Risikofaktoren (z.B. sicheres Trinkwasser, angemessene sanitäre Einrichtung) streng und objektiv definieren. Die Zusammenhänge zwischen Wasser, sanitären Einrichtungen, menschlicher Hygiene und Verhaltensweisen und der Schistosomen-Infektionen müssen ebenfalls besser erforscht werden.

***Übersetzung:*** David Croll
